# Family-Centered Prevention Effects on the Association Between Racial Discrimination and Mental Health in Black Adolescents: Secondary Analysis of 2 Randomized Clinical Trials

**DOI:** 10.1001/jamanetworkopen.2021.1964

**Published:** 2021-03-24

**Authors:** Gene H. Brody, Tianyi Yu, Edith Chen, Gregory E. Miller, Allen W. Barton, Steven M. Kogan

**Affiliations:** 1 Center for Family Research, University of Georgia, Athens; 2 Department of Psychology and Institute for Public Policy, Northwestern University, Evanston, Illinois; 3 Department of Human Development and Family Studies, University of Illinois, Champagne-Urbana; 4 Department of Human Development and Family Science and Center for Family Research, University of Georgia, Athens

## Abstract

**Importance:**

Some Black adolescents who frequently experience racial discrimination develop mental health problems. Protective caregiving may buffer adolescents from the negative mental health outcomes associated with experiencing racial discrimination.

**Objective:**

To examine if participation in programs that enhance protective caregiving will attenuate the positive association between Black adolescents’ encounters with discrimination and subsequent increases in mental health problems.

**Design, Setting, and Participants:**

This secondary analysis used data from 2 randomized clinical trials testing family-centered prevention programs: the Strong African American Families–Teen (SAAF–T) program and the Adults in the Making (AIM) program. The programs were implemented in community locations convenient for participants in 12 rural Georgia counties. For the SAAF–T trial, Black adolescents and their primary caregivers were recruited from 2007 to 2008. In the AIM trial Black adolescents and their primary caregivers were recruited from 2006 to 2007. Data for this study were analyzed from June to August 2020.

**Exposures:**

Adolescents provided data at baseline on the frequency of their encounters with racial discrimination. Treatment group participants in each trial took part in a family-centered prevention program designed to prevent substance use and mental health problems. SAAF–T is a 5-session, 10-hour psychosocial intervention for families with a Black adolescent aged 14 to 16 years. AIM is a 6-session, 12-hour psychosocial intervention for families with a Black youth who is a high school senior.

**Main Outcomes and Measures:**

The primary outcomes were mental health problems, including conduct problems and depression or anxiety symptoms.

**Results:**

The SAAF–T study included 502 Black adolescents (mean [SD] age, 16.0 [0.6] years; 281 [56.0%] girls), including 252 randomized to the intervention and 250 randomized to the control, and the AIM trial included 367 Black adolescents (mean [SD] age, 17.7 [0.8] years; 217 [59.1%] girls and women), including 187 randomized to the intervention and 180 randomized to the control. Adolescents assigned to the SAAF–T intervention group who frequently experienced discrimination at baseline evinced fewer subsequent increases in conduct problems (incident risk ratio, 0.530 [95% CI, 0.340 to 0.783]). Adolescents assigned to the AIM intervention group who frequently experienced discrimination at baseline evinced fewer subsequent increases in conduct problems (mean difference, −0.361 [95% CI, −0.577 to −0.144]) and fewer subsequent increases in depression or anxiety symptoms (mean difference, −0.220 [95% CI −0.402 to −0.038]). Moderated mediation analyses suggested that enhanced protective caregiving was partially responsible for all observed interaction effects (indirect effect: SAAF-T conduct problems, −0.063 [95% CI, −0.127 to −0.001]; AIM conduct problems, −0.048 [95% CI, −0.095 to −0.001]; AIM depression or anxious symptoms, −0.036 [95% CI, −0.074 to 0]).

**Conclusions and Relevance:**

This secondary analysis of 2 randomized clinical trials found that participation in family-centered preventive interventions attenuated the association between frequent exposure to discriminatory behaviors and subsequent mental health problems. Notably, all but 1 of the treatment and moderated-mediation findings were reproduced across the SAAF–T and AIM trials.

**Trial Registrations:**

ClinicalTrials.gov Identifiers: SAAF–T, NCT04501471; AIM, NCT04510116

## Key Points

**Question:**

Is participation in a family-centered prevention program, designed to enhance caregiving practices, associated with protection of Black adolescents from the effects of racial discrimination on their mental health?

**Findings:**

This secondary analyses of data from 2 randomized clinical trials found that participation in a family-centered prevention program was associated with protection of Black adolescents from the effects of racial discrimination on conduct problems (in both trials) and on depression/anxiety symptoms (in 1 trial). These associations were partially explained by intervention-induced changes in protective parenting.

**Meaning:**

These findings suggest that family-centered prevention programs reduce the effects of racial discrimination on subsequent increases in mental health problems among Black adolescents.

## Teaser

This secondary analysis of 2 randomized clinical trials examines the effects of family-centered prevention programs in mediating the adverse mental health outcomes associated with experience of racial discrimination among Black youths.

## Introduction

Racial discrimination includes routine experiences with disrespect and treatment connoting that one is inferior. It is pervasive in Black youth’s everyday lives.^1,2^ Children and adolescents who encounter racial discrimination are at risk for poor mental health outcomes, such as hopelessness, conduct problems, drug use, and depression.^3^ Importantly, not all Black youth exposed to racial discrimination go on to develop mental health problems.^3^ This observation raises a fundamental question for pediatric scientists studying resilience and for clinical researchers developing applications: what enables some Black youth to remain mentally healthy despite experiences with discrimination? Research suggests that receipt of protective caregiving, defined as caregiving that includes high levels of emotional support and warmth, involvement, bidirectional communication, and cooperative problem solving, may mitigate mental health problems associated with discrimination.^4,5,6^

However, causal inferences cannot be made on the basis of existing observational studies. In this study, we used secondary data from 2 randomized clinical trials of family-centered interventions to investigate the protective effects of caregiving practices. The Strong African American–Teen (SAAF–T)^7^ program was designed for youth aged 14 to 16 years, and the Adults in the Making (AIM)^8^ program was designed for youth aged 17 to 18 years. Both programs were developed to prevent mental health problems and substance use by enhancing protective caregiving. Previous research with the SAAF–T and AIM trials focused on establishing their efficacy and durability in preventing substance use and mental health problems.^9^ Both interventions have also been shown to mitigate the effects of life stress on Black adolescents by increasing protective caregiving, and both programs have demonstrated stress-buffering capacities for a range of psychosocial outcomes.^9^ However, these interventions’ effects on youth’s coping with discrimination have not been investigated.

Consistent with prior research that has documented larger intervention effects for those at higher risk,^10^ we hypothesized that SAAF–T and AIM would produce the greatest mental health benefits for adolescents who reported more frequent discrimination at baseline. Specifically, we hypothesized that adolescents assigned to the SAAF–T or AIM intervention group who reported frequent discrimination at baseline would evince fewer subsequent increases in mental health problems than would adolescents assigned to a control group. Finally, we explored the possibility that SAAF–T and AIM effects in reducing the impact of discrimination on youth’s mental health were attributable to improved protective caregiving.

## Methods

All procedures in SAAF–T and AIM were approved by the University of Georgia institutional review board. Adult participants provided written informed consent for themselves and their children; children provided written assent. Both trials follow the Consolidated Standards of Reporting Trials (CONSORT) reporting guideline. Trials were registered retrospectively because registration of trials with behavioral interventions and outcomes was not common at the time of the trials’ initiation.

### Study Design

We conducted an unplanned secondary analysis of data from 2 community-based randomized clinical trials, SAAF–T and AIM. Both trials were unblinded, parallel studies. In each trial, families were randomly allocated on a 1:1 ratio to intervention or control groups by the study statistician. Additional trial details are provided in the trial protocol and statistical analysis plan in Supplement 1.

### Participants

#### SAAF–T

**Figure 1.**
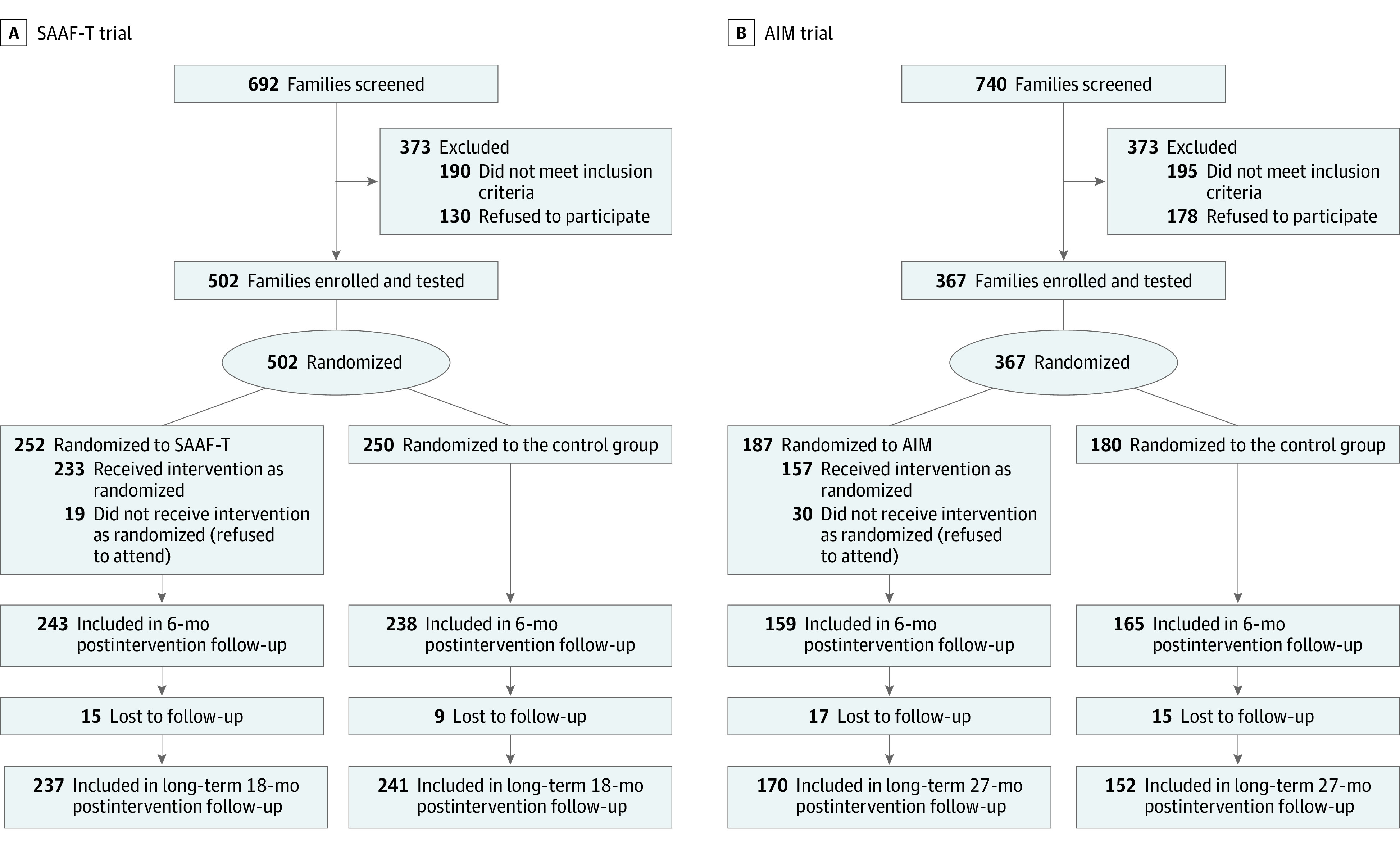
Participant Flowcharts AIM indicates Adults in the Making; SAAF–T, Strong African American Families–Teen.

Informed by a power analysis (Statistical Analysis Plan in Supplement 1), the SAAF–T trial included Black families from rural Georgia recruited from 2006 to 2007. In each family, an adolescent who was aged 16 years at recruitment and the adolescent’s primary caregiver (in most families, the biological mother) provided data. Schools in 6 counties provided lists of 10th-grade students, from which participants were selected randomly. Eligible youth were in the 10th grade and self-reported African American or Black race/ethnicity. Youth or caregivers with developmental disabilities or psychiatric illnesses that prevented them from completing data collection or participating in the intervention were excluded. Figure 1 presents the flow of participants at each stage of the trial.

#### AIM

Per power analysis (Statistical Analysis Plan in Supplement 1), AIM participants were Black youth and their primary caregivers. They resided in 6 rural Georgia counties; none of these counties were included in the SAAF–T trial. Schools in the counties from which the participants were recruited provided lists of 12th-grade students. Eligible youth were in the 12th grade and self-reported African American or Black race/ethnicity. Youth or caregivers with developmental disabilities or psychiatric illnesses that prevented them from completing data collection or participating in the intervention were excluded. Figure 1 presents the flow of participants at each stage of the trial.

### Intervention Implementation

SAAF–T consisted of 5 consecutive weekly, 2-hour sessions held at community facilities, with separate skill-building curricula for youth and primary caregivers.^9^ Caregivers were taught emotional and instrumental support, limit setting, adaptive racial socialization, and methods for communicating about sex and alcohol use. Youth learned the importance of abiding by household rules, setting goals for the future and making plans to attain them, and strategies for resisting substance use. Each meeting included a 1-hour session when youth and parents met separately, followed by a 1-hour parent-youth session during which participants practiced the skills they had learned in their separate sessions.

AIM provided support for developmentally appropriate caregiving before adolescents leave high school and assume occupational and continuing educational roles. Youth and their primary caregivers attended 6 consecutive weekly meetings and, as in SAAF–T, separate parent and youth skill-building curricula during each 2-hour session. Primary caregivers were taught protective emotional and instrumental support, occupational and educational mentoring, cooperative problem solving, and communication skills. Youth were taught how to make plans to meet their goals, to identify people in their communities who could help them with goal attainment, and to formulate self-care strategies.

### Data Collection Procedures

All data were collected in participants’ homes using standardized protocols. Interviews were conducted privately, with no other family members present or able to overhear the conversation. Families were compensated $100 at each wave of data collection. Families in both trials provided baseline data 2 months before the intervention (in 2006 for AIM and in 2007 for SAAF–T), postintervention data 6 months after baseline, and long-term follow-up data 18 months after baseline for SAAF–T (in 2010) and 27 months after baseline for AIM (in 2010).

### Measures

#### Racial Discrimination

At baseline, adolescents in both trials completed the Schedule of Racist Events,^11^ which has been used frequently in longitudinal studies with adolescents.^3^ Items assessed the frequency with which adolescents encountered discriminatory events, such as racial slurs, physical threats, and false accusations (SAAF–T: α = .90; AIM: α = .86).

#### Adolescent Mental Health

For measures of adolescent mental health in SAAF–T, adolescents responded to the Conduct Problems Scale from the National Youth Survey.^12^ Internal consistency analyses were not conducted because this instrument yields count data. In AIM, conduct problems were measured using the parents’ reports on the Child Behavioral Checklist.^13^ Responses to the Aggression and Rule Breaking subscales were summed to form an indicator of conduct problems ( baseline, α = .85; long-term follow-up, α = .89). In SAAF–T, depressive symptoms were assessed using adolescents’ reports on the Center for Epidemiologic Studies–Depression scale^14^ (baseline, α = .82; long-term follow-up, α = .83). In AIM, parents reported on youths’ depression or anxiety symptoms with the Child Behavioral Checklist (baseline, α = .83; long-term follow-up, α = .86).

#### Protective Caregiving

Before and after participation in prevention programming, SAAF–T and AIM primary caregivers reported the frequency and quality of their protective caregiving practices. In SAAF–T, protective caregiving was assessed with the Family Support Inventory^15^ and the Discussion Quality Scale.^16^ The Family Support Inventory measured emotional support, involvement, and quality of caregiver-youth communication (baseline, α = .85; postintervention, α = .94). The Discussion Quality Scale assessed communication frequency and quality for discussions of difficult issues (baseline, α = .77; postintervention, α = .79). Ratings on the 2 scales were correlated at *r* = .452 at baseline and *r* = .386 at postintervention (*P* < .001), so the ratings were standardized and summed to form a SAAF–T protective caregiving index. For AIM, frequency of protective caregiving was assessed using a caregiver-report questionnaire developed for research with Black families.^17^ The items assessed the extent to which the primary caregiver provided emotional support, was accessible to the youth, and discussed difficult issues with which the youth was dealing (baseline, α = .72; postintervention, α = .76).

#### Socioeconomic Risk Index

A socioeconomic risk index with 6 dichotomous variables was used as a control in the data analyses. A score of 1 was assigned to each of the following: family poverty (based on federal guidelines), caregiver unemployment, receipt of Temporary Assistance for Needy Families, caregiver single parenthood, caregiver education level less than high school graduation, and caregiver-reported inadequacy of family income. The scores were summed to form an index ranging from 0 to 6, with higher scores indicating more socioeconomic risk.

#### Intervention Status and Sex

Intervention participants were coded 1, and control participants were coded 0. Boys and men were coded 1, and girls and women were coded 0.

### Statistical Analysis

Intent-to-treat analyses conducted with Mplus statistical software version 8.2 (Muthén & Muthén) used the full information maximum likelihood estimator, which tests hypotheses against all available data. Thus, missing data did not result in exclusion. A logarithmic transformation was performed on the Center for Epidemiologic Studies–Depression scale and the Child Behavioral Checklist, effectively approximating normal distributions. Baseline equivalence between experimental conditions was examined for socioeconomic risk, participant sex, protective caregiving, and mental health outcomes using *t* tests.

Study hypotheses were tested with regression models in which mental health outcomes were estimated from baseline levels of mental health variables, discrimination, intervention condition, and the discrimination × intervention condition interaction term. Sex and socioeconomic risk were controlled in all analyses. Linear regression was used except for conduct problems in SAAF–T, where Poisson regression better fit the data.

Intervention-induced changes in protective caregiving were examined with regression-based moderated mediation models.^18^ First, we determined whether participation in SAAF–T or AIM improved protective caregiving for youth who frequently encountered discrimination (path A). Second, we calculated regression coefficients reflecting the associations between improvements in protective caregiving and mental health outcomes (path B). Moderated mediation was examined using data from steps 1 and 2. To do this, path A × path B regression coefficients were calculated.

*P* values were 2-sided, and statistical significance was set at .05. Data were analyzed from June to August 2020.

## Results

The SAAF–T study included 502 Black adolescents (mean [SD] age, 16.0 [0.6] years; 281 [56.0%] girls), including 252 randomized to the intervention and 250 randomized to the control, and the AIM trial included 367 Black adolescents (mean [SD] age, 17.7 [0.8] years; 217 [59.1%] girls and women), including 187 randomized to the intervention and 180 randomized to the control. Although the caregivers in the SAAF–T group worked a mean (SD) of 41.5 (20.4) hours per week, 320 families (63.8%) lived below federal poverty standards, and another 91 families (18.1%) lived within 150% of the poverty threshold. Similarly, in the AIM group, caregivers worked a mean (SD) of 38.5 (11.1) hours per week, but 153 families (41.7%) lived below federal poverty standards, and another 60 families (16.3%) lived within 150% of the poverty threshold. SAAF–T families attended a mean (SD) of 4 (1.5) of 5 total sessions, and 126 AIM families (67.3%) took part in 4 or more sessions, with 65 (34.8%) attending all 6 sessions. Study measures’ correlations and descriptive statistics can be found in eTable 1 and eTable 2 in Supplement 2.

### Sample Equivalence at Baseline and Attrition

Baseline equivalence was established on socioeconomic risk, participant sex, protective caregiving, and mental health outcomes (Table 1). Evaluation of baseline equivalence on study variables for participants who did or did not provide follow-up data × prevention group assignment revealed no significant main effects or interaction effects for any covariate or study variable.

**Table 1.** Baseline Characteristics of SAAF–T and AIM Participants

Characteristic	Mean (SD)	
	Intervention	Control
**SAAF-T**		
No.	252	250
Boys, No. (%)	112 (44.4)	109 (43.6)
Family socioeconomic risk	2.42 (1.42)	2.22 (1.43)
Encountered discrimination	−0.01 (0.97)	0.01 (1.03)
Protective caregiving	0.08 (1.65)	−0.08 (1.76)
Conduct problems	4.83 (0.33)	5.52 (0.38)
Depressive symptoms	13.81 (8.65)	13.80 (8.76)
**AIM**		
No.	187	180
Boys and men, No. (%)	69 (36.9)	81 (45.0)
Family socioeconomic risk	2.03 (1.33)	2.00 (1.46)
Encountered discrimination	−0.06 (1.00)	0.06 (1.00)
Protective caregiving	53.19 (5.24)	53.49 (6.16)
Conduct problems	4.68 (4.40)	4.05 (4.16)
Depressive/anxious symptoms	2.63 (3.18)	2.40 (2.96)

Abbreviations: AIM, Adults in the Making; SAAF–T, Strong African American Families–Teen.

### Discrimination, Intervention Participation, and Adolescent Mental Health

**Figure 2.**
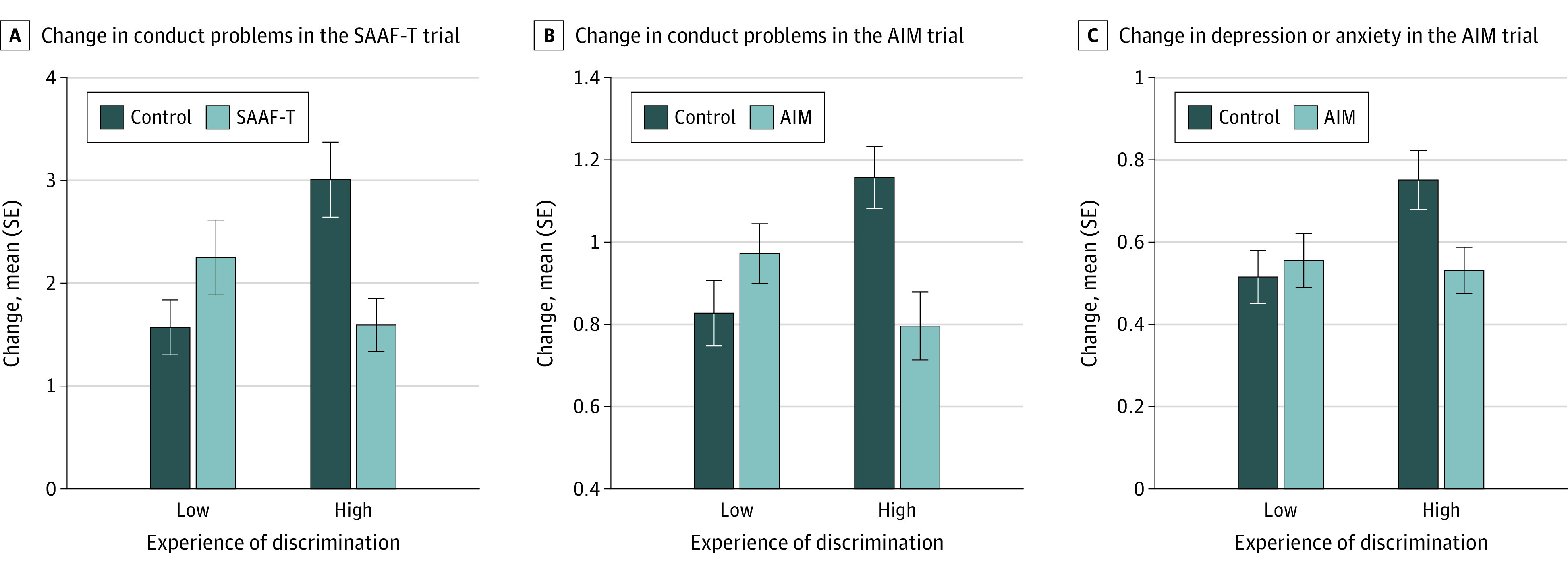
Estimated Means of Changes in Adolescents’ Conduct Problems and Depression or Anxiety Symptoms AIM indicates Adults in the Making; SAAF–T, Strong African American Families–Teen; low discrimination, 1-SD decrease; high discrimination, 1-SD increase; error bars, 1 SE.

Consistent with our hypotheses, significant discrimination × prevention status interactions emerged for 3 of 4 outcomes: conduct problems in the SAAF–T trial (Table 2), and conduct problems and depression or anxiety symptoms in the AIM trial (Table 3). Adolescents assigned to the SAAF–T and AIM interventions who experienced frequent discrimination evinced fewer increases in conduct problems (SAAF–T: incident risk ratio, 0.530 [95% CI, 0.340 to 0.783]; AIM: mean difference, −0.361 [95% CI, −0.577 to −0.144]), and, for AIM, adolescents in the intervention group who experienced frequent discrimination evinced fewer increases in depression or anxiety symptoms (mean difference, −0.220 [95% CI, −0.402 to −0.038]) than did similar youth assigned to a control group. No differences emerged between adolescents in the intervention or control groups when exposure to discrimination was low (SAAF–T conduct problems: incident risk ratio, 1.433 [95% CI, 0.910 to 2.257]; AIM conduct problems: mean difference, 0.145 [95% CI, −0.068 to 0.358]; AIM depression/anxiety: mean difference, 0.040 [95% CI, −0.141 to 0.221]). Figure 2 presents mental health outcomes at lower (1-SD below the mean) and higher (1-SD above the mean) levels of discrimination. Youth sex was not associated with moderation of any of the results.

**Table 2.** Racial Discrimination and Intervention Status as Risk Factors for Conduct Problems and Depressive Symptoms at the Long-term Follow-up in the SAAF–T Group

Factor	*b* (95% CI)	
	Conduct problems	Depressive symptoms
Male sex	.258 (−.042 to .558)	.028 (−.077 to .133)
Baseline family socioeconomic risk	.078 (−.032 to .187)	.032 (−.004 to .069)
Baseline conduct problems	.058 (.048 to .068)	NA
Baseline depressive symptoms	NA	.496 (.405 to .588)
Baseline encountered discrimination	.325 (.164 to .485)	.033 (−.037 to .104)
Intervention	−.137 (−.457 to .183)	−.107 (−.209 to −.005)
Encountered discrimination × SAAF–T intervention	−.497 (−.772 to −.221)	−.038 (−.140 to .063)

Abbreviations: NA, not applicable; SAAF–T, Strong African American Families–Teen.

**Table 3.** Racial Discrimination and Intervention Status as Risk Factors for Conduct Problems and Depressive/Anxious Symptoms at the Long-term Follow-up in the AIM Group

Factor	*b* (95% CI)	
	Conduct problems	Depressive/anxious symptoms
Male sex	−.013 (−.167 to .140)	−.043 (−.173 to .086)
Baseline family socioeconomic risk	.037 (−.018 to .092)	.026 (−.020 to .073)
Baseline conduct problems	.583 (.489 to .677)	NA
Baseline depressive/anxious symptoms	NA	.567 (.482 to .652)
Baseline encountered discrimination	.165 (.056 to .274)	.118 (.025 to .210)
Intervention	−.108 (−.260 to .044)	−.090 (−.218 to .038)
Encountered discrimination × AIM intervention	−.253 (−.404 to −.102)	−.130 (−.257 to −.002)

Abbreviations: AIM, Adults in the Making; NA, not applicable.

### Intervention-Induced Increases in Protective Caregiving and SAAF–T and AIM Effects on Mental Health Outcomes

We explored the hypothesis that the SAAF–T and AIM prevention effects for youth who encountered frequent discrimination were associated with improvements in protective caregiving. After controlling for youth sex, family socioeconomic risk, and baseline levels of protective caregiving and youth variables, regression models indicated a significant racial discrimination × intervention condition interaction in estimating postintervention levels of protective caregiving (eTables 3-6 in Supplement 2). The eFigure in Supplement 2 shows that primary caregivers in both SAAF–T and AIM evinced significant improvements in protective caregiving when youth experienced frequent discrimination (SAAF–T: mean difference, 0.429 [95% CI, 0.164 to 0.694]; AIM: mean difference, 1.664 [95% CI, 0.458 to 2.870]). The second set of regression models found improvements in protective caregiving to be negatively associated with conduct problems among both SAAF–T participants (*b* = −0.148 [95% CI, −0.266 to −0.030]) and AIM participants (*b* = −0.029 [95% CI, −0.049 to −0.009]) and with depression or anxiety symptoms among AIM participants (*b* = −0.022 [95% CI, −0.039 to −0.006]) (eTables 3-6 in Supplement 2). The results of moderated mediation analyses indicate that the association of the AIM intervention with reductions in depression or anxiety symptoms among youth who frequently encountered discrimination were completely mediated by protective caregiving (indirect effect: −0.036 [95% CI, −0.074 to 0]) (eTable 7 in Supplement 2). For conduct problems, better outcomes in this domain among youth in SAAF–T and AIM who frequently experienced discrimination were partially but not completely attributable to improvements in protective caregiving (indirect effect: SAAF-T conduct problems, −0.063 [95% CI, −0.127 to −0.001]; AIM conduct problems, −0.048 [95% CI, −0.095 to −0.001]; AIM depression or anxious symptoms, −0.036 [95% CI, −0.074 to 0]). These patterns indicate that SAAF–T and AIM were associated with reduced conduct problems through a combination of protective caregiving and additional pathways that have not yet been identified.

## Discussion

We analyzed data from 2 randomized clinical trials, including participants with similar demographic characteristics, that tested family-centered interventions. The programs were structured similarly, but were designed for different age groups (early high school vs high school seniors) and featured distinct content. The findings suggest that family-centered prevention attenuated associations between racial discrimination and subsequent increases in mental health problems. Youth who received the SAAF–T or AIM intervention who also encountered increased racial discrimination had significantly fewer conduct problems 1.5 to 2 years after the intervention than did youth in the control groups. Also, AIM participants who received the intervention and who experienced racial discrimination more frequently exhibited fewer increases in depressive or anxiety symptoms than did participants in the control group. However, this pattern did not hold for SAAF–T participants and depressive symptoms. This may be because the AIM participants were older than the SAAF–T participants, so AIM participants may have been at increased risk for exposure to racial discrimination than were the younger SAAF–T participants. Therefore, AIM participants may have benefited more from the increases in protective caregiving they received in the course of the prevention trial than did the participants in SAAF–T.

Moderated-mediation analyses were consistent with a scenario in which both SAAF–T and AIM reduced symptoms of mental health problems, in part, by enhancing the protective caregiving that youth received. The mediation analyses suggested that more supportive parents may be better able to establish strategies that enhance their children’s emotion regulation for coping with racial discrimination. This in turn may have reduced the physiological and psychological effects of racial discrimination that can influence mental health.^19,20^ To our knowledge, this is the first study to show that family-centered prevention was associated with buffering the effects of racial discrimination on adolescents’ mental health. It is notable that nearly all of the findings in this study were reproduced across the 2 randomized trials, thus increasing confidence in the results.

These results suggest that the buffering influences of enhanced protective caregiving on mental health outcomes are likely to be robust. In that regard, the results also converge with findings in the pediatric literature suggesting that a significant proportion of youth develop resilience to the mental and physical health consequences of adversity if they receive protective caregiving.^21^ Clinically, the findings suggest that SAAF–T, AIM, and perhaps other interventions that focus on strengthening protective caregiving could help to forestall or attenuate some of the mental health problems that racial discrimination can produce. Of particular relevance to pediatric clinical practice, efficacious family-centered prevention programs designed to enhance protective caregiving are available for Black preadolescents, adolescents, and youth who are about to transition to young adulthood.^9^ Participation in these programs has been associated with stress-buffering effects on the development of self-control and on reductions in drug use, obesity, cytokine levels, epigenetic aging, and, in this study, mental health problems following discrimination.^9^

### Limitations

This study has some limitations, including a lack of data on the effectiveness of SAAF–T and AIM outside of the areas in the rural southern United States where they were developed. Their efficacy in urban settings must be evaluated. AIM used parent report for both mediation and outcome variables, thus potentially increasing associations owing to common method bias. The trials concluded 10 years ago, and the historical context is evolving rapidly; additional replication with more recent trials is needed. Because the trials examined the durability of intervention effects, we were not able to provide the intervention to control participants. It is important that future research uses designs to provide control group participants with the opportunity to experience the intervention, particularly when they are from historically underrepresented and exploited populations. The availability of such designs in prevention science is currently limited.

## Conclusions

The findings of this secondary analysis of 2 randomized clinical trials underscore the susceptibility of adolescents who frequently encounter racial discrimination to elevated mental health symptoms. Secondary data analyses of 2 randomized trials suggest that supportive parenting may offset these mental health risks.

## Notes

Supplement 1. **Trial Protocol and Statistical Analysis Plan**

Supplement 2. **eTable 1.** Correlations and Descriptive Statistics Among Study Variables: SAAF–T **eTable 2.** Correlations and Descriptive Statistics Among Study Variables: AIM **eTable 3.** Changes in Protective Caregiving From Baseline to Postintervention and the Effects of Encountered Discrimination on Conduct Problems at Long-Term Follow-up by Intervention Status: SAAF–T **eTable 4.** Changes in Protective Caregiving From Baseline to Postintervention and the Effects of Encountered Discrimination on Depressive Symptoms at Long-Term Follow-up by Intervention Status: SAAF–T **eTable 5.** Changes in Protective Caregiving From Baseline to Postintervention and the Effects of Encountered Discrimination on Conduct Problems at Long-Term Follow-up by Intervention Status: AIM **eTable 6.** Changes in Protective Caregiving From Baseline to Postintervention and the Effects of Encountered Discrimination on Depressive/Anxious Symptoms at Long-Term Follow-up by Intervention Status: AIM **eTable 7.** Conditional Indirect Effects (Moderated Mediation Effects) for Low and High Encountered Discrimination Conditions **eFigure.** Estimated Means of Changes in Protective Caregiving From Baseline to Postintervention Conduct Problems and Depressive or Anxious Symptoms

Supplement 3. **Data Sharing Statement**
