# Supplementary material for: Family-Centered Prevention Effects on the Association Between Racial Discrimination and Mental Health in Black Adolescents: Secondary Analysis of 2 Randomized Clinical Trials
Source: JAMA Netw Open. 2021 Mar 24;4(3):e211964. doi: 10.1001/jamanetworkopen.2021.1964 (PMC7991970; doi:10.1001/jamanetworkopen.2021.1964)
Supplement: Supplement 1. — Trial Protocol and Statistical Analysis Plan [file jamanetwopen-e211964-s001.pdf]

Supplemental Appendix 1

Trials Protocols

SAAF-T

A. SPECIFIC AIMS

In this proposal, we request funding to conduct a randomized prevention trial to test the SAAF–HS program's efficacy. The sample will include ~500 rural African American families with a 10th-grade student, half of whom will be assigned randomly to a SAAF–HS and half to an attention-control group. Pre-intervention, post-intervention, and long-term follow-up assessments of adolescents' substance use and high-risk sexual behavior will be gathered from the entire sample.

Our specific aims are as follows:

1. To test the hypothesis that rural African American adolescents randomly assigned to participate in SAAF–HS, compared to attention-control participants, will demonstrate lower rates of substance use initiation, and that those who already use substances (e.g., cigarettes; alcohol, including binge drinking; marijuana and other illicit substances) will use them less frequently and consume lower quantities at each follow-up assessment.
2. To test the hypothesis that rural African American adolescents randomly assigned to participate in SAAF–HS, compared to attention-control participants, will demonstrate lower rates of high-risk sexual behavior (e.g., instances of unprotected sexual intercourse, multiple sexual partners) at each follow-up assessment.

D. RESEARCH METHODS

D.1. Participants

A random sample of 500 rural African American families with a son or daughter in the first semester of 10th grade in a public high school will participate in the study.

D.1.1. Eligibility, Random Assignment, and Contamination

Lists of all African American 10th-grade students will be obtained from each of the 6 participating high schools. The students on each school's list will be randomly ordered to determine the sequence in which families will be contacted to be screened for eligibility. A small number of students will be excluded from the pool of potential participants. If the primary caregiver or adolescent does not speak English, which is unlikely, the youth will be excluded. Twins and stepsiblings who both are in the 10th grade will be excluded because the inclusion of more than one participant from the same family introduces confounds that make interpretation difficult. Youth from families who have taken part in any of the investigators' other studies will also be excluded. Although this is unlikely because participants for the proposed study will not be recruited from counties that were sampled for previous research, some participants may have moved to one of the targeted counties.

Enrolled families will be assigned randomly to either the SAAF–HS or attention-control condition (see recruitment procedures, section D.1.2). All families will be followed across the course of the proposed study and assessed regardless of the number of SAAF–HS or attention-control sessions they do or do not attend (an intent-to-treat analysis); this includes youth who drop out of school during the study.

D.1.2. Family Recruitment and Retention Strategies

The recruitment process will be conducted in the following sequence: (1) a letter from the school principal will be mailed to parents/ guardians informing them of the study; (2) a community liaison will visit the families' homes to determine their eligibility; and (3) eligible families will be invited to participate, with active consent obtained from primary caregivers and active assent from adolescents.

D.1.2a. Principals' letter. Each primary caregiver of an African American 10-grade student who is selected randomly from the pool of potential participants will receive a letter from the school principal describing the study and the school's collaboration with the University of Georgia (UGA). Caregivers will be informed that they will be contacted by representatives associated with UGA (community liaisons), that they and their 10th-grade son or daughter may be invited to participate, and that participation is voluntary.

D.1.2b. Community liaisons' home visit. Community liaisons will schedule home visits to students' families based on the randomly permuted order of the school list. During this visit, the liaison will describe the study to

the caregiver and student and tell them that, based on their responses to the screening questions (English-speaking, no siblings in the same grade, no prior participation in investigators' research), they may be eligible to participate. Adolescents and caregivers who do not meet any of the exclusionary criteria will be invited to participate in the study and will be informed of its requirements: active consent from a primary caregiver for his or her own participation and for the adolescent's participation; active assent from the adolescent for his or her own participation (consent forms can be found in Appendix D); random assignment to either the prevention or attention-control group; completion of questionnaires three times during an 18-month period; and caregivers' and students' participation in the prevention or attention-control program. A family will be classified as refusing at this point if either the caregiver or student does not wish to take part in the study.

D.1.2c. Monetary incentives. At each pretest, posttest, and follow-up assessment in the proposed project, each adolescent will receive a $50 gift certificate to a local department store and each primary caregiver will receive a $100 honorarium.

D.1.2d. Retention. Following each data collection home visit and prevention session, community liaisons contact participants to inquire about the quality of the experience and to answer questions. Between data collections, liaisons call families on a regular basis and visit the homes of families whose telephone service has been interrupted. The families also provide contact information for three individuals who will always know their whereabouts; these lists are updated at each home visit. All families receive a semiannual newsletter that includes a stamped address correction form to facilitate the updating of address lists before post office forwarding orders expire.

D.2. Overview of the Prevention and Research Procedures

All prevention and attention-control families will participate in a pretest, a posttest, and a long-term follow-up assessment 9 months after the posttest. Both prevention and attention-control families will participate in a 5-week intervention. The posttest data collection will take place 3 months after the interventions conclude to allow any transitory immediate effects to dissipate.

D.3. SAAF–HS Program Implementation

D.3.1. Group Leaders

Teams of three persons will lead the prevention and attention-control groups, each of which will include 10 to 12 families. All group leaders will be African Americans with a minimum of a bachelor's degree in psychology, nursing, education, social work, or a related field. The investigators will prepare a leaders' guide that will describe all facets of the delivery of the SAAF–HS and the attention-control program. The leaders will receive 30 hours of training on content delivery in a structured group process format, implementation of specific curriculum activities, guided practice in delivering and pacing curriculum segments, and leader self-care. Didactic material, role-playing exercises, and modeling will be used to teach the protocol for each session. Prior to completion of training, group leaders must demonstrate mastery of the curriculum by scoring 100% on a written examination covering the session material. Leaders will meet with the prevention coordinator each week to review the previous week's sessions and prepare for those in the coming week.

D.3.2. Treatment Integrity

Standardized procedures will be followed to insure the prevention sessions' integrity. The intervention manual includes a detailed format to be used for each session. Each team will be provided with materials designed to facilitate correct execution of the session protocol, including an outline of each session, a checklist of the materials necessary for each activity, the specific theme of each task, and forms for in-session notes. Presentation of targeted processes on videotapes also will help to ensure the intervention's integrity. The field supervisor also will make unannounced direct observations of the sessions. To assess program fidelity, all sessions will be videotaped and scored for curriculum coverage and protocol adherence using instruments developed for SAAF. Two judges will score one of every four sessions to assess interrater reliability (kappas).

D.3.3. Logistics

A meeting site will be established in each county at a community facility in a central location that is easily accessible to program participants. Churches and schools provide ample space for classrooms, waiting areas, and parking.

D.3.4. Engagement Procedures

Several strategies from SAAF will be used in the proposed program to address scheduling conflicts, transportation needs, and child care requirements. During the pretest, family members will be asked about their weekly schedules and sessions will be planned to accommodate the schedules of as many families as

possible. During the first half hour of each meeting, dinner will be served to family members attending the session. Leaders will arrange transportation for any families who need it and care will be provided for any children who come with the families. An intensive reminder protocol also will be used throughout the program. Community liaisons will call all participants before the program begins and prior to each weekly session to encourage their involvement. Reminder postcards will be sent from the CFR each week, highlighting the focus of the upcoming meeting. A community liaison will call participants who miss a meeting to remind them of the next meeting and address any barriers to attendance. In SAAF, these strategies resulted in attendance rates that were higher than those of middle-class European Americans participating in a family-centered substance use prevention program (Spoth et al., 2001).

D.4. SAAF–HS Program Structure and Content

The proposed intervention will be structured according to the SAAF delivery system. The prevention program will consist of seven consecutive weekly meetings, with separate caregiver and adolescent skill-building curricula and a family curriculum. Each meeting will include separate, concurrent sessions for caregivers and adolescents, followed by joint sessions during which the adolescents and their caregivers review, discuss, and practice the skills they learned in their separate sessions. Videotapes portraying family interactions and intrapersonal processes will be used in all sessions to ensure consistent presentation of key points. Each session will include a homework review, didactic presentation, group discussion, and skill-building activities that reinforce learning. The concurrent and family sessions will each last 1 hour; thus, adolescents and caregivers will receive 14 hours of prevention training. This dosage corresponds to Tobler's (2000) recommendations, based on a meta-analysis, for dosage levels needed to promote changes in intervention participants' behavior.

The complete SAAF–HS curriculum is presented in Appendix A. The program content is based on the causative model presented in Section B.5. The caregivers' curriculum focuses on enhancing developmentally appropriate, competence-promoting parenting strategies; promoting adolescents' racial identity and providing youth with strategies for dealing with discrimination; supporting academic engagement; providing information about HIV/AIDS and other sexually transmitted diseases; and communicating expectations about high-risk behavior. The adolescents' curriculum will address developing future orientations and formulating plans for continuing education and employment; enhancing academic engagement; developing problem-focused coping and adaptive strategies for dealing with discrimination; enhancing youths' ability to implement protective behaviors should they decide to engage in sexual activity; and understanding the effects of substance use in undermining protective sexual behavior.

D.5. Attention-control Condition Structure and Content

Participants randomly assigned to the attention-control condition will participate in a 7-week family-centered program designed to promote healthful behaviors among adolescents by encouraging good nutrition, exercise, and informed consumer behavior. The structure of the attention-control program will be identical to that of the SAAF–HS program: Each weekly meeting will include separate, concurrent 1-hour caregiver and youth sessions followed by a 1-hour family session, providing 14 hours of training. The content of the attention-control condition will be taken from the FUEL™ program, which includes four units for parents and youth. The first unit, Skin Deep, addresses media literacy, self-esteem, and body image, with an emphasis on consumer education. Energy for Action, the second unit, focuses on strategies for establishing a healthful diet. The third unit, The Perfect Machine, explains the benefits of physical activity and suggests strategies for incorporating it into the youths' daily lives. Taking on the World, the fourth unit, deals with positive activism for promoting healthful lifestyles in the youths' communities. Like SAAF–HS, FUEL™ includes videotaped exemplars of targeted behaviors and processes. During project year 1, Dr. Tracy Anderson, the CFR curriculum developer, will adapt the FUEL™ structure to make it identical to that of SAAF–HS. The procedures for training facilitators, ensuring intervention integrity, and engaging participants will also be equivalent to those used in the SAAF–HS implementation.

D.6. Data Collection

One 2-hour home visit will be made to each family for data collection at the pretest, posttest, and long-term follow-up assessments. To minimize cultural bias, African American students and community members, many of whom have worked at the CFR for many years, will serve as assessment assistants.

D.6.1. Intervention Assessment

Computer-assisted self-administered interviews using audio enhancements (ACASI) will be used to collect data from youth and caregivers. The interview measures will be administered via ACASI with the guidance of Assessment Assistants. The assistants, who will be blind to families' assignment to the SAAF–HS or attention-control condition, will arrange home visits with participants. The assistants will set up a computer for each family member at places in the residence that the family designates as private, then enter the participants' ID numbers and the commands necessary to begin the appropriate instruments. After leading the participants through a series of warm-up exercises and verifying that they are comfortable using the computers, the Assessment Assistants will remain available to help participants upon request. When data are returned to the CFR, the project coordinator will immediately check their completeness and integrity and ensure the ID numbers' accuracy. Within 8 business hours of data collection, a computer specialist will upload the data to a secure server; all data are backed up to a storage area network on an hourly basis.

D.6.1a. Training of Assessment Assistants. Assessment Assistants will be selected based on their previous experience with collecting data and interviewing adolescents about sensitive topics. Prior to administering the baseline assessment, they will receive 20 hours of intensive training and supervised practice that will address building rapport, using the ACASI system, and helping participants to use the system. Assistants also will meet each week with the Project Director to discuss any problems that arise. Prior to the follow-up assessments, training updates will be conducted in which any changes to the interview items or assessment procedures will be reviewed.

D.6.2. Process Evaluation of the Prevention Program

Using session attendance and program fidelity data (see section D.3.2), a dosage score will be calculated for each study participant. Both parents in two-parent families will be encouraged to attend each session of the program; in single-parent families, the parent and an extended family member who assists with the youth's upbringing will be encouraged to attend. If two adults cannot come, attendance of one will be encouraged as preferable to attendance of neither.

D.7. Measures

We will use multi-informant, multiple indicator strategies to assess the constructs underlying the intervention theory. Potential time-varying factors and risk factors that could compromise SAAF–HS effects will also be measured: parental depression, parental substance use, changes in parental relationships or employment situations, and personal life stressors. Including these variables in the longitudinal assessment will enable the investigators to determine whether the risk factors compromise the prevention program's efficacy or, conversely, the prevention program reduces the risk factors' impact. The measures can be found in Appendix E.

D.7.1. Demographics

The demographic interview was designed and tested in Georgia with samples of single mothers and married parents. Primary caregivers will report their yearly income, per capita income, employment status, duration of unemployment during the past 2 years, hours worked per week, education level, numbers of children and adults in the household, and relations of all household members to one another.

D.7.2. Competence-Promoting Parenting and Responsive Family Relationships

Five indicators comprise the parenting construct: involved-vigilant parenting; supportive, affectively positive family relationships; communication and expectations about risk behavior; parental involvement in adolescents' academic pursuits; and adaptive racial socialization.

D.7.2a. Involved-vigilant parenting. Adolescents and caregivers will report involved-vigilant parenting using an instrument from Brody and Murry's longitudinal, developmental research with rural African American families (Brody, Ge, et al., 2001; 2003). The scale's 19 items assess parental involvement, inductive discipline, consistent discipline, and monitoring. Responses to the items are summed. Cronbach's alphas exceed .70 (Brody, Murry, Gerrard, et al., 2004).

D.7.2b. Supportive, affectively positive family relationships. Adolescents and caregivers will assess support in their family relationships with the Family Support Inventory (Wills et al., 2003), a14-item measure of perceived availability of emotional and instrumental support. Internal consistency ranged from .82 to .88 for emotional support and .76 to .83 for instrumental support for rural African Americans caregivers and adolescents. The scales consistently predict low substance use, high self-regulation, and resilient coping (Wills et al., 2000, 2003). Four items that Carver, Scheier, and Weintraub (1989) developed will be used to assess the frequency with which family members provide emotional support. Cronbach's alphas in Brody and Murry's prior

prevention research ranged from .78 to .87 (Brody, Murry, Gerrard, et al., 2004). The Interaction Behavior Questionnaire (Prinz et al., 1979) assesses affective involvement and positivity in family communication. Cronbach's alphas for this scale with one of Brody and Murry's longitudinal research samples were .85 for caregivers and .79 for youths (Brody, Murry et al., 2002). The Discussion Quality Scale (Brody & Flor, 1998) will be used to assess parent-adolescent discussions regarding school, peers, alcohol, other drugs, and sex. For each topic, caregivers and adolescents are asked about the frequency of conversations, the relative contributions of parent and child to the conversations, and the frequency with which discussions become arguments. In Brody and Murry's prior research, Cronbach's alphas were .84 for caregivers and .79 for adolescents.

D.7.2c. Communication and expectations about risk behavior. Adolescents and caregivers will complete a 6-item scale that assesses parents' communication of expectations about substance use and sexual behavior (Spoth, Redmond, & Shin, 1998). Three items, which concern clear parental expectations about the behavior in question and association with peers who engage in it, are rated twice, once regarding substance use and once regarding sexual behavior. Parents' communication about sexual risk behavior also will be assessed with a 5-item scale that DiClemente and colleagues developed for use with African Americans. Adolescents and parents report the frequently with which they communicated about pregnancy prevention, STD prevention, HIV prevention, and condoms during the past 6 months. This measure was internally consistent ($\alpha = .88$) and associated with condom self-efficacy. The Process of Sexual Communication Scale (Brody, Murry, Gerrard, et al., 2004), used with youth in SAAF, assesses the quality of parent-child conversations regarding sex from caregivers' and adolescents' perspectives. Reliabilities exceeded .70 for both parent and youth reports.

D.7.2d. Parental involvement in youths' academic pursuits. Parental involvement in youths' schooling will be assessed from caregivers' and adolescents' perspectives using parallel versions of a 15-item scale that Brody et al. (1995) developed to assess parent participation in school-related activities. Cronbach's alpha for this scale exceeded .80. Adolescents and caregivers also will complete parallel versions of a 9-item scale adapted from the National Educational Longitudinal Study of 1988 (Ingels et al., 1994). The measure assesses parents' supervision of homework and discussions of academic planning. Internal consistency for this scale with 9th-grade students was .79

***D.7.2e. Adaptive racial socialization*.** Adolescents and caregivers will complete Stevenson's (1997) Scale of Racial Socialization for Adolescents (SORS-A). The measure includes 4 subscales that assess various aspects of adaptive racial socialization. Cultural Pride Reinforcement indexes encouragement to take pride in African American culture; Racism Awareness Teaching involves helping adolescents to be alert to racism and its consequences; Life Achievement Struggling emphasizes overcoming obstacles to achievement; and Extended Family Caring measures relatives' caregiving support

D.7.3. Adolescent Stressors

***D.7.3a. Racism*.** Adolescents, friends, and caregivers will report their experiences with racism using the Schedule of Racist Events (SRE; Landrine & Klonoff, 1996). We will use the SRE's Frequency of Unfair Treatment Based on Race and Stress of Racial Unfairness subscales. Respondents rate the occurrence and stressfulness of racially based unfair treatment by school officials, business employees, legal officials, neighbors, church members, friends, and strangers. In developmental research, Cronbach's alphas for youth and caregivers exceeded .75 for both subscales (Brody, Chen, et al., in press).

D.7.3b. Educational and occupational opportunities. The Total Resources subscale ($\alpha = .95$) from the Community Problems and Resources Scale (Forehand & Brody, 2000) will be used to assess the availability of 19 competence-promoting community characteristics such as job training, educational facilities, social organizations, and transportation. For the proposed study, additional items were developed to target educational and job opportunities.

D.7.4. Risk Factors and Time-Varying Life Circumstances with Potential Influence on SAAF–HS Efficacy

D.7.4a. Financial stress. Caregivers will report chronic financial difficulties using scales developed for Brody and Murry's longitudinal research (Brody, Ge, et al., 2001, 2003): Unmet Material Needs, Can't Make Ends Meet, Financial Adjustments, and Negative Financial Events. These scales' reliabilities with rural African American samples range from .69 to .80.

D.7.4b. Caregivers' life stress. Caregivers will report the stressors they encountered during the previous year, such as divorce or separation, violent victimization of a family member, and incarceration of a family

member (Kim, Brody, et al., 2005).

***D.7.4c. Caregiver depression*.** The Center for Epidemiologic Studies Depression Scale (Radloff, 1977), which was designed for administration to community samples, will be used to assess caregiver depression. Internal consistency with rural Georgia samples averages .84 (Brody, Murry, et al., 2002).

***D.7.4d. Caregiver substance use*.** Caregivers will report their own substance use on the Parent Alcohol/ Drug Use History (Collins, Parks, & Marlatt, 1985). Items assess drinking in a typical week and both lifetime and recent use of marijuana, stimulants, sedatives, psychedelic drugs, cocaine, heroin, and opiates. Internal consistencies for caregivers in Brody and Murry's prevention research ranged from .79 to .81.

D.7.4e. Caregiver employment and relationship status. Caregivers' reports of changes in employment, marital status, or cohabitation will be included in each year's demographic interview.

D.7.5. Negative Emotions

Adolescents and caregivers will report their negative emotions using the Depression, General Anxiety, and Hostility subscales of the Brief Symptom Inventory (Derogatis & Melisaratos, 1983), a measure of generalized distress (Piersma, Boes, & Reaume, 1994) that detects low-level negative emotions in community samples. Brody and Ge (2001) reported Cronbach's alphas exceeding .75 for rural African American youth and adults.

D.7.6. Adolescent Protective Mediators

D.7.6a. Future orientation. Adolescents will report their time orientations using a version of Zimbardo's Time Perspective Inventory (Zimbardo & Boyd, 1999) that Wills and colleagues adapted for use with multiethnic samples of adolescents (Wills, Sandy, & Yaeger, 2001). The Future subscale assesses the formulation of goals and plans to achieve them (α =. 77); the Present subscale indexes an orientation toward present enjoyment, pleasure, and excitement (α =. 79). Time perspective is associated with self-regulation, anger-based coping, and self-esteem (Wills et al., 2001). Adolescents and their friends also will use a scale developed for SAAF to report their ability to set, sustain, and achieve future-oriented goals. Cronbach's alpha at SAAF pretest was .72, and the scale was sensitive to program effects (Brody, Murry, Gerrard, et al., 2004).

***D.7.6b. Self-regulation*.** Adolescents will complete the Self-Control Inventory (Wills & Stoolmiller, 2002), which includes 25 items distributed among seven subscales (Soothability, Dependability, Planning, Problem Solving, Impatience, Distractibility, and Angerability). Wills and associates (Wills et al., 2000, 2003) reported reliabilities of .73 to .83 with rural African American adolescent samples. Caregivers will complete the Self-control and Lack of Self-control subscales of the Self-Control Scale (Humphrey, 1982), which Brody and Murry have used extensively with African American children and adolescents (Brody, Murry et al., 2002; Brody et al., 2004). The subscales assess goal setting, formulation of plans to attain goals, and persistence or lack thereof. Cronbach's alphas consistently exceed .75.

D.7.6c. Emotion regulation. Adolescents will report their coping styles using Wills and Hirky's (1996) Coping Processes Inventory. Respondents are presented with definitions of various coping behaviors and asked how often they use each approach for dealing with problems concerning school, parents, health, and sadness. Internal consistencies in a multiethnic sample of adolescents (Wills et al., 2002) exceeded .78. The proposed project will include the measure's anger-based, helpless, substance use, and avoidant coping subscales.

D.7.6d. Racial identity. Adolescents will report their racial identity using the Centrality and Regard subscales of the Multidimensional Model of Racial Identity for African Americans (Sellers et al., 1997). The investigators used these scales with African American youth in previous prevention research (Murry et al., 2005). Centrality (α = .70) indicates the importance of respondents' race to their self-definitions and Regard (α = .73) refers to affective and evaluative perceptions of one's race.

***D.7.6e. Academic engagement*.** Adolescents will report their academic engagement using a 20-item scale that Brody developed for use with rural African American youth; Cronbach's alpha = .90 (Brody, Ge et al., 2003).

D.7.6f. Negative attitudes about substance use and high-risk sexual behavior. Adolescents will report their own attitudes about substance use and high-risk sexual behavior using a scale that Brody and colleagues (Brody, Murry, Gerrard, et al., 2004) developed for rural African American youth. Reliabilities exceeded .80.

D.7.6h. Resistance efficacy. Adolescents will report their substance use resistance efficacy with the Drinking Refusal Self-Efficacy Questionnaire (DRSEQ; Young, Oei, & Crook, 1991). The DRSEQ has demonstrated internal consistency and construct validity and is associated with substance use quantity (Lee & Oei, 1993;Young & Oei, 2000).

D.7.6g. HIV prevention knowledge**.** Adolescents will report their HIV knowledge using a scale that DiClemente and Wingood developed in their ongoing research with African American adolescents (DiClemente et al., 2004). The 23-item scale uses a True-False-Don't Know response set. Correct responses are summed to create a composite score. Internal consistency is .86.

D7.6h. Expectancies and attitudes about sexually self-protective behavior**.** Adolescents will report their expectations about condom use on a version of the Condom Attitude Scale (St. Lawrence, Reitman, Jefferson, & Alleyne, 1994) adapted for administration to teens with low literacy. The internal consistency of the scale with African American adolescents participating in the SiHLE trial was .83. Outcome expectancies for communicating with a sexual partner about HIV-risk-related behavior will be assessed with the Sexual Communication Attitude Scale, a measure that DiClemente and Wingood developed to assess communication between adolescent sexual partners. This revised scale will be pilot tested and its psychometrics properties established prior to its use in the proposed research program.

D.7.6i. Self-efficacy for sexually protective behavior**.** Adolescents will report their self-efficacy for condom use on an 11-item scale that Wingood and DiClemente developed to measure African American adolescents' beliefs about their ability to use condoms effectively. The scale has an internal consistency of .84 (DiClemente et al., 2004). Adolescents and their friends also will report their confidence in communicating with partners about protective sexual behavior using a 7-item scale that Wingood and DiClemente developed. The scale demonstrated satisfactory internal consistency in the SiHLE study ($\alpha = .82$).

D.7.7. Adolescents' Outcomes

D.7.7a. Substance use. The substance use assessment is designed to address its multidimensional nature (Newcomb, 1992). From three well-validated measures, items were selected for their sensitivity to intervention effects at several levels of substance use. At the initiation level, group differences in the proportion of participants who transition from nonuse to use during the study can be detected. At the escalation level, group differences in the proportion of participants who increase their frequency/intensity of use during the study can be assessed. At the problem use level, group differences in specific substance-related behaviors and problems can be determined. The basic items in the measure are those included in the Monitoring the Future study (Johnston et al., 2004) that assess lifetime, annual, and 30-day prevalence and frequency of cigarette smoking, alcohol use, binge drinking, and marijuana use. For the purposes of the proposed study, a 6-month assessment at the long-term follow-up will be added. A checklist of other drugs includes lifetime use of LSD/acid, MDMA/Ecstasy, cocaine, heroin, other opiates, amphetamines, non-prescribed tranquilizers, and inhalants. Problems associated with substance use will be indexed using items from the Minnesota Student Survey for high school students (Harrison, Fulkerson, & Beebe, 1998) These scales include items assessing dependence (e.g., had to use more to get same effect, tried to cut down but couldn't) and negative consequences of use (e.g., use hurt relationships with friends or family, use interfered with other activities, use resulted in problems with law, missed school or work because of use).

D.7.7b. High-risk sexual behavior. The primary outcome for this variable is the proportion of vaginal and anal sexual acts in which condoms were used during the 30 days prior to the ACASI assessment. This measure is derived by dividing the number of protected encounters by the total number of encounters. This ratio is one of the most commonly used measures of outcome efficacy in HIV prevention trials (Celentano et al., 2002; Chesney et al., 2003; Gordon, Forsyth, Stall, & Cheever, 2005; Hobfoll et al., 2002; Rosser et al., 2002). Other self-reported sexual behaviors to be assessed include the number of unprotected vaginal and anal sexual encounters during the previous 3 months and condom use during the most recent instance of sexual intercourse. Information on number and types (primary, casual, new) of sexual partners; frequency of oral, anal, and vaginal sex; use of alcohol and other drugs prior to sexual intercourse; STD infection; pregnancy; and partners' risk status (e.g., sexual partner has other partners) will also be gathered.

Researchers widely recognize that participants' reports of their sexual practices might be inaccurate, either unintentionally or intentionally. We will take several steps to increase the self-report measures' validity. To minimize inaccurate recall, only sexual behavior that occurred during a relatively brief time period (i.e. during the past 30 days, during the past 3 months, and at last sexual intercourse) will be addressed. Participants also will be given a calendar on which the relevant dates are clearly marked. ACASI methodology also has been demonstrated to increase respondents' willingness to provide frank responses to sensitive questions about their sexual behavior (Macalino et al., 2002).

D.7.7c. Deviant behavior. Theoretically, antisocial behavior is related to substance use and high-risk sexual activity (Jessor, 1991), perhaps through a common cause such as parents' ineffective monitoring and discipline (Leigh & Stall, 1993). Adolescents will use a checklist from the National Youth Survey (Elliott, Ageton, & Huizinga, 1985) to report their own deviant behavior during the past 3 months and the proportion of their friends who engaged in the same behavior during that time period.

D.8. Data Analysis

D.8.1. Data Management, Exploratory Data Analysis, Missing Data, and Psychometrics

Data will be organized and cleaned following quality control procedures developed in the SAAF trial, which involve checking for outlying values and inappropriate patterns of missing data. Next, items' distributions and constructs' psychometric properties will be examined. Both exploratory and confirmatory factor analyses will be conducted, using data transformations and factor analysis with ordinal scales where the data's distributions warrant it (Muthén & Muthén, 2004). Mplus (Muthén & Muthén, 2004), a comprehensive structural equation and growth curve modeling program, will be used to examine the stability of the scales' factor structure across the pretest, posttest, and long-term follow-up assessments. Mplus permits construction of sophisticated structural equation models and handles missing data using full maximum likelihood methods in which all available data are included under a "missing at random" assumption. These solutions will be compared to models that allow certain types of non-ignorably missing data to assess the stability of these assumptions (Brown, Indurkhya & Kellam, 2000). Standard exploratory data analytic methods will be used to examine departures from normality and to determine the type of modeling that is required. For example, substance use and sexual behavior variables typically include large proportions of abstainers along with a skewed distribution of engagement in high-risk behavior. These types of data, which routinely have been analyzed by creation of abstinent/nonabstinent dichotomies or treated as continuous normal-like variables, will be examined using more complex two-part models (Olsen & Schafer, 2001) that allow separate tracking of the intervention's impact on abstinence/ nonabstinence and levels of risk behavior. Finally, for many of the analyses of intervention-targeted behaviors and attitudes, confirmatory factor analyses will be used when possible to form latent constructs by combining data from different informants. If the informants' perspectives do not converge enough to make this possible, each informant's data will be included in the analyses as a separate variable.

D.8.2. Analyses for Aims 1 and 2: Primary Outcomes

The primary issue to be addressed in Aims 1 and 2 is SAAF–HS's effectiveness in deterring the onset and escalation of substance use and high-risk sexual behavior across the 18 months separating the pretest and long-term follow-up assessments. A dramatic growth in substance use and high-risk sexual behavior is likely to occur across measurement points, which include the beginning of 10th grade, middle of 10th grade, and beginning of 11th grade. SAAF–HS's impact on each of the outcomes will be examined in three ways: on posttest (middle of 10th grade), on long-term follow-up (beginning of 11th grade), and on growth trajectories. The two single-time-point analyses will be conducted with Structural Equation Modeling (SEM) using the maximum likelihood method and controlling for baseline levels of substance use and high risk-sexual behavior. The first outcome analysis (posttest) identifies reduction in substance use and high risk-sexual behavior; the second (long-term follow-up) will be used for tests of delayed initiation and reduction of substance use and reduction of high-risk sexual behavior. We will explore, in an additional analysis, SAAF–HS's effects on delaying the onset of sexual intercourse. SAAF–HS, however, is likely to have its strongest impact on growth trajectories (Taylor et al., 2000). A baseline growth model with random intercepts and slopes will be established for the attention-control condition. A multigroup analysis will then be conducted, testing the equivalence of growth parameters between the attention-control and the SAAF–HS groups (Byrne, 2001). If the multigroup analyses detect differences between the slopes (rates of growth for substance use or high-risk sexual behavior) of the SAAF–HS and attention-control group, impact statements will be made by examining how the average substance use and sexual risk behavior trajectories change with time.

The three-time-point design allows one degree of freedom in fitting linear growth patterns. An exploratory analysis will be conducted to assess nonlinearity in growth over time (Wang, Brown & Bandeen-Roche, 2005). If these diagnostic patterns indicate nonlinearity, the fit will be improved through transformation of the time axis by freeing the last loading, effectively incorporating nonlinearity. Separate analyses will be used to examine impact on alcohol, tobacco, and marijuana use because different intervention impacts occasionally emerge for different substances. In all these models, fit will be compared using formal likelihood ratio statistics, Wald-type tests, and diagnostic techniques that Brown and colleagues developed (Wang, Brown & Bandeen-Roche,

2005). For analyses of high-risk sexual behavior, two outcomes, unprotected sex at last encounter and number of unprotected encounters in the past 3 months, will be analyzed using binary outcomes and Poisson counts, respectively. For each of these repeated measures, we anticipate two types of "zeros": one indicating abstinence and another indicating condom use. Two approaches will be used, one that incorporates a separate model of abstinence and condom use over time as a function of intervention condition and another that models these outcomes as "zero-inflated" Binomial and Poisson models, similar to Carlin et al.'s (2002) modeling of smoking behavior.

D.8.3. Analysis of Aims 3 and 4: Changes in Intervention-targeted Behaviors

Aims 3 and 4 focus on SAAF–HS's impact on competence-promoting and responsive family relationships and protective adolescent mediators. These aims will be analyzed using the same methods used for aims 1 and 2. SAAF–HS's impact on the outcome variables will be analyzed with posttest, long-term follow-up, and growth trajectory data. The two single-time-point analyses will be conducted using SEM, controlling for baseline levels of the outcome variables. Again, SAAF–HS should evince it strongest impact on growth trajectories. The same method used to analyze growth trajectories for Aims 1 and 2 will be used for Aims 3 and 4.

D.8.6. Statistical Power

In this section, we discuss statistical power for the models we described previously, including the SAAF–HS intervention's impact on changes in the slopes of drug use and high-risk sexual behavior and on outcomes at a single point in time. In these calculations, we assume conservatively a 13% loss in data across time, yielding two groups of 250 participants in each condition. For growth models with either continuous or discrete outcomes, we have used power calculations that Brown and colleagues have made available on the internet (http://psmg.usf.edu/products.html) to determine the smallest effect size we can detect with 80% power for changes in the slope parameter, assuming linear growth over three time points. When the measurement reliability is 60%, we can achieve 80% power when the ES is 0.1 at posttest and grows to 0.2 at long-term follow-up. For conducting a test at a single time point on a continuous outcome, based on Cohen (1988), we can detect an effect size of 0.25 at 80% for a 0.05 level test, assuming negligible variation based on school. We also have calculated power by taking this factor into account. Based on variability observed in other preventive trials among schools and individuals (Brown and Liao, 1999) the power is 80% to detect an ES of 0.36 and 70% to detect an ES of 0.31 for 0.05 level tests. Thus, we clearly have adequate power to detect a moderate effect for continuous outcomes involving single time points. For a discrete measure at a single time point, such as a comparison of the proportion of individuals who are using a substance, we will be able to detect an odds ratio of at least 1.6 with 80% power using a 0.05 level chi-square test (Fleiss et al., 2003). This odds ratio corresponds to a reduction from 50% users to 38% in the intervention group.

G. LITERATURE CITED

Achenbach, T. M. (1991). *Manual for the Child Behavior Checklist/ 4-18 and 1991 profile*. Burlington, VT: University of Vermont Department of Psychiatry.

Agnew, R. (2001). Building on the foundation of general stress theory: Specifying the types of strain most likely to lead to crime and delinquency. *Journal of Research in Crime and Delinquency, 38*, 319-361.

Allen, L., & Majidi-Ahi, S. (1989). Black American children. In J. T. G. Huang & N. L (Eds.), *Children of color* (pp. 148-178). San Francisco: Jossey-Bass.

Anderson, E. (1990). *Streetwise: Race, class, and change in an urban community*. Chicago: University of Chicago Press.

Andrews, J. A., & Duncan, S. C. (1997). Examining the reciprocal relation between academic motivation and substance use: Effects of family relationships, self-esteem, and general deviance. *Journal of Behavioral Medicine, 20*, 523-549.

Andrews, J. A., Tildesley, E., Hops, H., & Li, F. (2002). The influence of peers on young adult substance use. *Health Psychology, 21*(4), 349-357.

Arnett, J. J. (2000). Emerging adulthood: A theory of development from the late teens through the twenties. *American Psychologist, 55*, 469-480.

Bachanas, P. J., Morris, M. K., Lewis-Gess, J. K., Sarett-Cuasay, E. J., Sirl, K., Ries, J. K., et al. (2002). Predictors of risky sexual behavior in African American girls: Implications for preventive interventions. *Journal of Pediatric Psychology, 27*, 519-530.

Baldwin, C., Baldwin, A., & Cole, R. (1990). Stress resistant families and stress resistant children. In J. Rolf, A. Masten, D. Cicchetti, K. Nuechterlein & S. Weintraub (Eds.), *Risk and protective factors in the development of psychopathology* (pp. 257-280). New York: Cambridge University Press.

Bandura, A. (1997). *Self-efficacy: The exercise of control*. New York: Freeman.

Bane, M. J., & Ellwood, D. T. (1989). One fifth of the nation's children: Why are they poor? *Science, 245*, 1047-1053.

Barkley, R. A. (1997). Behavioral inhibition, sustained attention, and executive functions: Constructing a unifying theory of ADHD. *Psychological Bulletin, 121*, 65-94.

Bauman, K. E., & Ennett, S. T. (1994). Peer influence on adolescent drug use. *American Psychologist, 49*, 820-822.

Baumeister, R. F., & Sher, S. J. (1988). Self-defeating behavior patterns among normal individuals: Review and analysis of common self-destructive tendencies. *Psychological Bulletin, 104*, 3-22.

Benda, B. B., & Corwyn, R. F. (1996). Testing a theoretical model of adolescent sexual behavior among rural families in poverty. *Child & Adolescent Social Work Journal, 13*(6), 469-494.

Biafora, F., & Zimmerman, R. (1998). Developmental patterns of African-American adolescent drug use. In W. A. Vega & A. G. Gil (Eds.), *Drug use and ethnicity in early adolescence*. New York: Plenum Press.

Billy, J. O. G., Brewster, K. L., & Grady, W. R. (1994). Contextual effects of the sexual behavior of adolescent women. *Journal of Marriage & the Family, 56*(2), 387-404.

Boatright, S. R. (2003). *The Georgia County Guide: 2003*. Athens, GA: Center for Agribusiness and Economic Development.

Bollen, K. A. (1989). *Structural equations with latent variables*. New York:: Wiley.

Brody, G. H., Chen, Y.-f., Murry, V. M., Ge, X., Simons, R. L., Gibbons, F. X., et al. (in press). Perceived discrimination and the adjustment of African American youths: A five-year longitudinal analysis with contextual moderation effects. *Child Development*.

Brody, G. H., Dorsey, S., Forehand, R., & Armistead, L. (2002). Unique and protective contributions of parenting and classroom processes to the adjustment of African American children living in single-parent families. *Child Development, 73*, 274-286.

Brody, G. H., & Flor, D. L. (1996). Coparenting, family interactions, and competence among African American youths. In J. P. McHale & P. A. Cowan (Eds.), *Understanding how family-level dynamics affect children's development: Studies of two-parent families* (pp. 77-91). San Francisco, CA: Jossey Bass.

Brody, G. H., & Flor, D. L. (1998). Maternal resources, parenting practices, and child competence in rural, single-parent African American Families. *Child Development, 69*, 803-816.

Brody, G. H., Flor, D. L., Hollett-Wright, N., & McCoy, J. K. (1998). Children's development of alcohol use norms: Contributions of parent and sibling norms, children's temperaments, and parent-child discussion. *Journal of Family Psychology, 12*, 209-219.

Brody, G. H., Flor, D. L., & Neubaum, E. (1998). Coparenting processes and child competence among rural African American families. In M. Lewis & C. Feiring (Eds.), *Families, risk, and competence* (pp. 227-243). Mahwah, NJ: Erlbaum.

Brody, G. H., & Forehand, R. (1985). The efficacy of parent training with maritally distressed and non-distressed mothers: A multimethod assessment. *Behaviour Research and Therapy, 23*, 291-296.

Brody, G. H., & Ge, X. (2001). Linking parenting processes and self-regulation to psychological functioning and alcohol use during early adolescence. *Journal of Family Psychology, 15*, 82-94.

Brody, G. H., Ge, X., Conger, R., Gibbons, F. X., Murry, V. M., Gerrard, M., et al. (2001). The influence of neighborhood disadvantage, collective socialization, and parenting on African American children's affiliation with deviant peers. *Child Development, 72*, 1231-1246.

Brody, G. H., Ge, X., Katz, J., & Arias, I. (2000). A longitudinal analysis of internalization of parental alcohol-use norms and adolescent alcohol use. *Applied Developmental Science, 4*(2), 71-79.

Brody, G. H., Ge, X., Kim, S. Y., Murry, V. M., Simons, R. L., Gibbons, F. X., et al. (2003). Neighborhood disadvantage moderates associations of parenting and older sibling problem attitudes and behavior with conduct disorders in African American children. *Journal of Consulting & Clinical Psychology, 71*.

Brody, G. H., Jack, L., McBride-Murry, V., Landers-Potts, M., & Liburd, L. (2001). Heuristic model linking contextual processes to self-management in African American adults with Type 2 Diabetes. *Diabetes Educator, 27*(5), 17-25.

Brody, G. H., Kim, S., Murry, V. M., & Brown, A. C. (2003). Longitudinal direct and indirect pathways linking older sibling competence to the development of younger sibling competence. *Developmental Psychology, 39*, 618-628.

Brody, G. H., Kim, S., Murry, V. M., & Brown, A. C. (2004). Protective longitudinal paths linking child competence to behavioral problems among African American siblings. *Child Development, 75*, 455-467.

Brody, G. H., Kim, S., Murry, V. M., & Brown, A. C. (2005). Longitudinal links among parenting, self-presentations to peers, and the development of externalizing and internalizing symptoms in African American siblings. *Development and Psychopathology, 17*, 185-205.

Brody, G. H., Murry, V. M., Brown, A. C., & Anderson, T. (2004, May, 2004). *Translating research into prevention effects.* Paper presented at the Society for Prevention Research, Montreal, Canada.

Brody, G. H., Murry, V. M., Gerrard, M., Gibbons, F. X., McNair, L., Brown, A. C., et al. (in press). The Strong African American Families Program: Prevention of high-risk behaviors and a test of a model of change. *Journal of Family Psychology*.

Brody, G. H., Murry, V. M., Gerrard, M., Gibbons, F. X., Molgaard, V., McNair, L., et al. (2004). The Strong African American Families Program: Translating research into prevention programming. *Child Development, 75*, 900-917.

Brody, G. H., Murry, V. M., Kim, S., & Brown, A. C. (2002). Longitudinal pathways to competence and psychological adjustment among African American children living in rural single-parent households. *Child Development, 73*, 1505-1516.

Brody, G. H., Murry, V. M., Kogan, S. M., Gerrard, M., Gibbons, F. X., Brown, A. C., et al. (in press). The Strong African American Families Program: A cluster-randomized prevention trial of long-term effects and a mediational model. Manuscript submitted for publication. *Journal of Consulting & Clinical Psychology*.

Brody, G. H., Murry, V. M., McNair, L., Chen, Y., Gibbons, F. X., Gerrard, M., et al. (2005). Linking changes in parenting to parent-child relationship quality and youth self control: The Strong African American Families Program. *Journal of Research on Adolescence, 14*, 47-69.

Brody, G. H., Neubaum, E., Boyd, G. M., & Dufour, M. (1997). Health consequences of alcohol use in rural America. In E. B. Robertson, Z. Sloboda, G. M. Boyd, L. Beatty & N. J. Kozel (Eds.), *Rural substance abuse: State of knowledge and issues* (Vol. NIDA research monograph 168, pp. 250-363). Rockville, MD: National Institute of Drug Abuse.

Brody, G. H., Stoneman, Z., & Flor, D. (1994). Financial resources, parent psychological functioning, parent co-caregiving, and early adolescent competence in rural two-parent African American families. *Child Development, 65*(590-605).

Brody, G. H., Stoneman, Z., & Flor, D. (1995). Linking family processes and academic competence among rural African American youths. *Journal of Marriage & the Family, 57*, 567-579.

Brody, G. H., Stoneman, Z., Flor, D., McCrary, C., Hastings, L., & Conyers, O. (1994). Financial resources, parent psychological functioning, parent co-caregiving, and early adolescent competence in rural two-parent African-American families. *Child Development, 65*, 590-605.

Brody, G. H., Stoneman, Z., & MacKinnon, C. (1982). Role asymmetries in interactions between school-aged children, their younger siblings, and their friends. *Child Development, 53*, 1364-1370.

Brody, G. H., Stoneman, Z., & Wheatley, P. (1984). Peer interaction in the presence and absence of observers. *Child Development, 55*, 1425-1428.

Bronfenbrenner, U., & Morris, P. (1998). The ecology of developmental process. In R. M. Lerner (Ed.), *The handbook of child psychology* (Vol. I: Theory, pp. 993-1029). New York: John Wiley and Sons.

Brown, C. S., & Bigler, R. S. (2005). Children's perceptions of discrimination: A developmental model. *Child Development, 76*, 533-553.

Brown CH, Indurkhya A, and Kellam SG (2000). Power calculations for data missing by design with application to a follow-up study of exposure and attention. *Journal of the American Statistics Association, 95,* 383-395.

Brown, C. H., & Liao, J. (1999). Principles for designing randomized preventive trials in mental health: an emerging developmental epidemiology paradigm. *Am J Community Psychol, 27*, 673-710.

Bryant, A. L., Schulenberg, J., Bachman, J. G., O'Malley, P. M., & Johnston, L. D. (2000). Understanding the links among school misbehavior, academic achievement, and cigarette use: A national panel study of adolescents. *Prevention Science, 1*, 71-87.

Cairns, R., Cairns, B., Neckerman, H., Gest, S., & Gariepy, J. L. (1988). Social networks and aggressive behavior: Peer support or peer rejection? *Developmental Psychology, 24*, 815-823.

Capaldi, D., Chamberlain, P., Fetrow, R., & Wilson, J. E. (1997). Conducting ecologically valid prevention research: Recruiting and retaining a "whole village" in multimethod, multiagent studies. *American Journal of Community Psychology, 25*, 471-492.

Carlin, J.B., Wolfe, R., Brown, C.H., and Gelman, A. (2002). A case study on the choice, interpretation, and checking of multilevel models for longitudinal, binary outcomes. *Biostatistics*, *2*, 397-416.

Carver, C. S., Scheier, M. F., & Weintraub, J. K. (1989). Assessing coping strategies: A theoretically based approach. *Journal of Personality and Social Psychology, 56*, 267-283.

Catalano, R. F., Morrison, D. M., Wells, E. A., & Gilmore, M. R. (1992). Ethnic differences in family factors related to early drug initiation. *Journal of Studies on Alcohol, 53*, 208-217.

Celentano, D. D., DiIorio, C., Hartwell, T., Kelly, J., Magana, R., Maibach, E., et al. (2002). Predictors of sexual behavior patterns over one year among persons at high-risk for HIV. *Archives of Sexual Behavior, 31*(2), 165-176.

Center on Aids and Community Health. (2003). *Structural interventions HIV prevention and public health: Descriptive summary of selected literature*. Atlanta, GA: Academy for Educational Development.

Centers for Disease Control and Prevention. (1998). Risks for HIV infection among persons residing in rural areas and small cities: Selected sites, southern United States, 1995-1996. *Morbidity and Mortality Weekly Report, 47*(974-978).

Centers for Disease Control and Prevention. (2005, April 22, 2005). *HIV/AIDS Surveillance in Adolescents (through 2003)*. Retrieved August 15, 2005, 2005, from http://www.cdc.gov/hiv/graphics/adolesnt.htm

Centers for Disease Control and Prevention, H. A. P. R. S. P. (1999, Revised). *Compendium of HIV prevention interventions with evidence of effectiveness*. Atlanta, GA: Centers for Disease Control and Prevention.

Chassin, L., Presson, C. C., Pitts, S. C., & Sherman, S. J. (2000). The natural history of cigarette smoking from adolescence to adulthood in a Midwestern community sample: Multiple trajectories and their psychosocial correlates. *Health Psychology, 19*, 223-231.

Cheong, J. W., MacKinnon, D. P., & Khoo, S. T. (2003). Investigation of mediational processes using parallel process latent growth curve modeling. *Structural Equation Modeling, 10*(2), 238-262.

Chesney, M. A., Koblin, B. A., Barresi, P. J., Husnik, M. J., Celum, C. L., Colfax, G., et al. (2003). An individually tailored intervention for HIV prevention: Baseline data from the EXPLORE study. *American Journal of Public Health, 93*(6), 933-938.

Cicchetti, D., & Toth, S. L. (1992). The role of developmental theory in prevention and intervention. *Developmental Psychopathology, 4*, 489- 493.

Cohen, J. (1988). *Statistical power analysis for the social sciences*. Mahwah, NJ: Erlbaum.

Collins, R. L., Parks, G. A., & Marlatt, G. A. (1985). Social determinant of alcohol consumption: The effects of social interaction and model status on the self-administration of alcohol. *Journal of Consulting & Clinical Psychology, 53*, 189-200.

Conger, R. D., & Elder, G. H. (1994). *Families in troubled times: Adapting to changes in rural America*. New York: Aldine de Gruyter.

Cooksey, E. C., Menaghan, E. G., & Jekielek, S. M. (1997). Life-course effects of work and family circumstances on children. *Social Forces, 76*, 637-665.

Cooper, M. L., Pierce, R. S., & Huselid, R. F. (1994). Substance use and sexual risk taking among black adolescents and white adolescents. *Health Psychology, 13*, 251-262.

Corbie-Smith, C. G., Thomas, S. B., Williams, M. V., & Moody-Ayers, S. (1999). Attitudes and beliefs of African Americans toward participation in medical research. *Journal of General Internal Medicine, 14*, 537-546.

Crosby, R. A., DiClemente, R. J., Wingood, G. M., & Harrington, K. (2002). HIV/STD prevention benefits of living in supportive families: A prospective analysis of high-risk African-American female teens. *American Journal of Health Promotion, 16*(3), 142-145.

Crosby, R. A., DiClemente, R. J., Wingood, G. M., Lang, D. L., & Harrington, K. (2003). Infrequent parental monitoring predicts sexually transmitted infections among low-income African American female adolescents. *Archives of pediatrics & adolescent medicine., 157*(2), 169-173.

Crosby, R. A., Wingood, G. M., DiClemente, R. J., & Rose, E. S. (2002). Family-related correlates of sexually transmitted disease and barriers to care: a pilot study of pregnant African American adolescents. *Family and Community Health, 25*, 16-27.

Crosnoe, R. (2001). Academic orientation and parental involvement in education during high school. *Sociology of Education, 74*, 210-230.

Crowne, D. P., & Marlowe, D. (1960). A new scale of social desirability independent of psychopathology. *Journal of Consulting Psychology, 24*, 349-354.

Dane, A. V., & Schneider, B. H. (1998). Program integrity in primary and secondary prevention: Are implementation effects out of control? *Clinical Psychology Review, 18*, 23– 45.

Derogatis, L. R., & Melisaratos, N. (1983). The Brief Symptom Inventory: An introductory report. *Psychological Medicine, 13*(3), 595-605.

DiClemente, R. J., & Crosby, R. A. (2003). Sexually transmitted diseases among adolescents: risk factors, antecedents, and prevention strategies. In G. R. Adams & M. Berzonsky (Eds.), *The Blackwell Handbook of Adolescence* (pp. 573-605). Oxford, England: Blackwell Publishers Ltd.

DiClemente, R. J., & Wingood, G. M. (1995). A randomized controlled trial of an HIV sexual risk-reduction intervention for young African-American women. *JAMA: Journal of the American Medical Association, 274*, 1271-1276.

DiClemente, R. J., Wingood, G. M., Crosby, R. A., Cobb, B. K., Harrington, K., & Davies, S. L. (2001). Parent-adolescent communication and sexual risk behaviors among African American adolescent females. *The Journal of Pediatrics, 139*, 407-412.

DiClemente, R. J., & Crosby, R. A. (2003). Sexually transmitted diseases among adolescents: Risk factors, antecedents, and prevention strategies. In G. R. Adams & M. Berzonsky (Eds.), *The Blackwell Handbook of Adolescence* (pp. 573-605). Oxford, England: Blackwell.

DiClemente, R. J., Wingood, G. M., Crosby, R. A., Rose, E., Lang, D., Pillay, A., et al. (2004). A descriptive analysis of STD prevalence among urban pregnant African-American teens: Data from a pilot study. *Journal of Adolescent Health, 34*, 376-383.

DiClemente, R. J., Wingood, G. M., Crosby, R. A., Sionean, C., Brown, L. K., Rothbaum, B., et al. (2001). A prospective study of psychological distress and sexual risk behavior among black adolescent females. *Pediatrics, 108*, E85-90.

DiClemente, R. J., Wingood, G. M., Harrington, K. F., Lang, D. L., Davies, S. L., Hook, E. W., et al. (2004). Efficacy of an HIV prevention intervention for African American adolescent girls: A randomized controlled trial. *JAMA: Journal of the American Medical Association, 292*, 171-179.

Dishion, T. J., & Andrews, D. W. (1995). Preventing escalation in problem behaviors with high-risk young adolescents: Immediate and 1-year outcomes. *Journal of Consulting and Clinical Psychology, 63*, 538-548.

Dishion, T. J., Bullock, B. M., & Granic, I. (2002). Pragmatism in modeling peer influence: Dynamics, outcomes, and change processes. *Development and Psychopathology, 14,* 969-981.

Dishion, T. J., & Kavanagh, K. (2000). A multilevel approach to family-centered prevention in schools: Process and outcome. *Addictive Behaviors, 25*, 889-911.

Dishion, T. J., McCord, J., & Poulin, F. (1999). Iatrogenic effects that aggregate high-risk youth. *American Psychologist, 54*.

Dishion, T. J., & Patterson, G. R. (1999). Model building in developmental psychopathology: A pragmatic approach to understanding and intervention. *Journal of Clinical Child Psychology, 28*, 502-512.

Doljanac, R. F., & Zimmerman, M. A. (1998). Psychosocial factors and high-risk sexual behavior: Race differences among urban adolescents. *Journal of Behavioral Medicine, 21*(5), 451-467.

Donner, A., & Klar, N. (2004). Pitfalls of and controversies in cluster randomization trials. *American Journal of Public Health, 94*, 416-422.

DuRant, R. H., Smith, J. A., Kreiter, S. R., & Krowchuk, D. P. (1999). The relationship between early age of onset of initial substance use and engaging in multiple health risk behaviors among young adolescents. *Archives of Pediatric & Adolescent Medicine, 153*(3), 286-291.

Dusenbury, L., Brannigan, R., Falso, M., & Hansen, W. B. (2003). A review of research on fidelity of implementation: Implications for drug abuse prevention in school settings. *Health Education Research, 18*, 237-256.

Eisenberg, N., Fabes, R., Nyman, M., Bernzweig, J., & Pinuelas, A. (1994). The relations of emotionality and regulation to children's anger-related reactions. *Child Development, 65*, 109-128.

Elliott, D. S., Ageton, S. S., & Huizinga, D. (1985). *Explaining delinquency and drug use*. Beverly Hills, CA: Siegel.

Elliott, D. S., Ageton, S. S., Huizinga, D., Knowles, B. A., & Canter, R. J. (1983). *The prevalence and incidence of delinquent behavior: 1976-1980.* Boulder, CO: Behavioral Research Institute.

Fabes, R. A., & Eisenberg, N. (1992). Young children's coping with interpersonal anger. *Child Development, 63*, 116-128.

Fisher, C. B., Wallace, S. A., & Fenton, R. E. (2000). Discrimination distress during adolescence. *Journal of Youth & Adolescence, 29*, 679-695.

Flay, B. R., Allred, C. G., & Ordway, N. (2001). Effects of the Positive Action program on achievement and discipline: Two matched-control comparisons. *Prevention Science, 2*, 71-89.

Fleiss, J. L., Levin, B. & Paik, M. C. (2003). *Statistical methods for rates and proportions* (3rd ed). Hoboken, NJ: Wiley-Interscience.

Folkman, S., Chesney, M. A., Pollack, L., & Phillips, C. (1992). Stress, coping, and high-risk sexual behavior. *Health Psychology, 11*(4), 218-222.

Forehand, R., & Brody, G. H. (2000). The Role of community risks and resources in the psychosocial adjustment of at-risk children: An examination across two community contexts and two informants. *Behavior Therapy, 31*(3), 395.

Fortenberry, J. D. (1995). Adolescent substance use and sexually transmitted diseases risk: A review. *Journal of Adolescent Health, 16*, 304-308.

Friedman, A. S., Granick, S., Bransfield, S., Kreisher, C., & Schwartz, A. (1996). The consequences of drug use/abuse for vocational career: A longitudinal study of a male urban African-American sample. *American Journal of Drug and Alcohol Abuse, 22*, 57-73.

Fromme, K., & Rivet, K. (1994). Young adults' coping style as a predictor of their alcohol use and response to daily events. *Journal of Youth and Adolescence, 23*, 85-97.

Frone, M. R., Cooper, M. L., & Russell, M. (1994). Stressful life events, gender, and substance use: An application of Tobit regression. *Psychology of Addictive Behaviors, 8*, 59-69.

Furstenberg, F. F., Jr., Cook, T. D., Eccles, J., Elder, G. H., Jr., & Sameroff, A. (1998). *Managing to make it: Urban families and adolescent success.* Chicago: University of Chicago Press.

Gibbons, F. X., Gerrard, M., Cleveland, M. J., Wills, T. A., & Brody, G. (2004). Perceived discrimination and substance use in African American parents and their children: A panel study. *Journal of Personality and Social Psychology, 86*, 517-529.

Gibbons, F. X., Gerrard, M., Lune, L. S. V., Wills, T. A., Brody, G., & Conger, R. D. (2004). Context and cognitions: Environmental risk, social influence, and adolescent substance use. *Personality & Social Psychology Bulletin, 30*(8), 1048-1061.

Gibbons, F. X., Gerrard, M., Vande Lune, L. S., Wills, T. A., Brody, G., & Conger, R. D. (in press). Context and cognitions: Environmental risk, social influence, and adolescent substance use. *Personality & Social Psychology Bulletin*.

Gillespie, R. (1991). *Manufacturing knowledge: A history of the Hawthorne experiments*. Cambridge: Cambridge University Press.

Gordon, C. M., Forsyth, A. D., Stall, R., & Cheever, L. W. (2005). Prevention interventions with persons living with HIV/AIDS: State of the science and future directions. *AIDS Education & Prevention, 17*, 6-20.

Gore, S., & Aseltine, R. H., Jr. (2003). Race and ethnic differences in depressed mood following the transition from high school. *Journal of Health and Social Behavior, 44*, 370-390.

Griesler, P. C., Kandel, D. B., & Davies, M. (2002). Ethnic differences in predictors of initiation and persistence of adolescent cigarette smoking in the National Longitudinal Survey of Youth. *Nicotine and Tobacco Research, 4*, 79-93.

Grunbaum, J. A., Kann, L., Kinchen, S., Ross, J., Hawkins, J., Lowry, R., et al. (2004). Youth Risk Behavior Surveillance--United States, 2003. *Morbidity and Mortality Weekly Report, 53/SS-2*, 1-100.

Guilamo-Ramos, V., Litardo, H. A., & Jaccard, J. (2005). Prevention programs for reducing adolescent problem behaviors: Implications of the co-occurrence of problem behaviors in adolescence. *Journal of Adolescent Health, 36*, 82-86.

Guyll, M., Spoth, R., & Redmond, C. (2003). The effects of incentives and research requirements on participation rates for a community-based preventive intervention research study. *the Journal of Primary Prevention, 24*, 25-41.

Hall, H. I., Li, J., & McKenna, M. T. (2005). HIV in predominantly rural areas of the United States. *Journal of Rural Health, 21*, 245-254.

Hansen, W. B., Graham, J. W., Wolkenstein, B., & Rohrbach, L. (1991). Program integrity as a moderator of prevention program effectiveness: Results for fifth grade students in the Adolescent Alcohol Prevention Trial. *Journal of Studies on Alcohol, 52*, 568-579.

Hansen, W. B., Tobler, N. S., & Graham, J. W. (1990). Attrition in substance abuse prevention research: A meta-analysis of 85 longitudinally followed cohorts. *Evaluation Review, 14*, 677-685.

Harrison, P. A., Fulkerson, J. A., & Beebe, T. J. (1998). DSM-IV substance use disorder criteria for adolescents: A critical examination based on a statewide school survey. *The American Journal of Psychiatry, 155*, 486-492.

Hewitt, M. (2002). Attitudes toward interview mode and comparability of reporting sexual behavior by personal interview and audio computer-assisted self interviewing: Analyses of the 1995 National Survey of Family Growth. *Sociological Methods and Research, 31*, 3-26.

Hill, R. B. (1972). *The Strength of Black Families*. New York: Emerson Hall.

Hobfoll, S. E., Jackson, A. P., Lavin, J., Johnson, R. J., & Schrader, K. E. E. (2002). Effects and generalizability of communally oriented HIV-AIDS prevention versus general health promotion groups for single, inner-city women in urban clinics. *Journal of Consulting & Clinical Psychology, 70*(4), 950-960.

Holz, V. J., & Tienda, M. (1998). Education and employment in a diverse society: Generating inequality through the school to work transition. In N. Denton & S. Tolnay (Eds.), *American diversity: A demographic challenge for the twenty-first century* (pp. 249-281). Albany, NY: SUNY Press.

Hughes, D., & Chen, L. (1997). When and what parents tell children about race: An examination of race-related socialization among African American families. *Applied Developmental Science, 1*(4), 200-214.

Humphrey, L. L. (1982). Children's and teachers' perspectives on children's self control: The development of two rating scales. *Journal of Consulting & Clinical Psychology, 50*, 624-633.

Ingels, S. J., Dowd, K. L., Stipe, J. L., Bartot, V. H., & Frankel, M. R. (1994). *Second follow-up: Student component data file user's manual*. Retrieved May 15, 2004, from http://nces.ed.gov/pubs94/94374ch1.pdf

Institute of Medicine. (1994). *Reducing risks for mental disorders: Frontiers for preventative intervention research*. Washington, DC: National Academy Press.

Jessor, R. (1991). Risk behavior in adolescence: A psychosocial framework for understanding and action. *Journal of Adolescent Health, 12*, 597-605.

Johnston, L. D., O'Malley, P. M., & Bachman, J. G. (2003). *Monitoring the Future national survey results on drug use, 1975-2002. Volume I: Secondary school students* (Vol. NIH Publication No. 03-5375). Bethesda, MD: National Institute on Drug Abuse.

Johnston, L. D., O'Malley, P. M., Bachman, J. G., & Schulenberg, J. E. (2004). *Monitoring the Future national results on adolescent drug use: Overview of key findings, 2003*. Bethesda, MD: National Institute of Drug Abuse.

Kandel, D. B., & Raveis, V. H. (1989). Cessation of illicit drug use in young adulthood. *Archives of General Psychiatry, 46*, 109-116.

Kellam, S. G. (1990). Developmental epidemiological framework for family research on depression and aggression. In G. R. Patterson (Ed.), *Depression and aggression in family interaction.* (pp. 11-48): Lawrence Erlbaum Associates, Inc.

Kellam, S. G., & Anthony, J. C. (1998). Targeting early antecedents to prevent tobacco smoking: Findings from an epidemiologically based randomized field trial. *American Journal of public Health, 88*, 1490-1495.

Kirby, D. (2002). The impact of schools and school programs upon adolescent sexual behavior. *The Journal of Sex Research, 39*, 27-33.

Kogan, S. M., Berkel, C., Chen, Y., Brody, G. H., & Murry, V. M. (in press). Metro status and African American adolescents' risk for substance use. *Journal of Adolescent Health*.

Kogan, S. M., Luo, Z., Murry, V. M., & Brody, G. H. (in press). Risk and protective factors predicting substance use among African American dropouts. *Psychology of Addictive Behaviors*.

Kotchick, B. A., Dorsey, S., Miller, K. S., & Forehand, R. (1999). Adolescent sexual risk-taking behavior in single-parent ethnic minority families. *Journal of Family Psychology, 13*, 93-102.

Kraemer, H. C., Wilson, G. T., Fairburn, C. G., & Agras, W. S. (2002). Mediators and moderators of treatment effects in randomized clinical trials. *Archives of General Psychiatry, 59*, 877-883.

Kumpfer, K. L., & Alvarado, R. (1995). Strengthening families to prevent drug use in multiethnic youth. In G. Botvin, S. Schinke & M. Orlandi (Eds.), *Drug Abuse Prevention with Multiethnic Youth* (pp. 253-292). Newbury Park, CA: Sage Publications.

Kumpfer, K. L., & Alvarado, R. (2003). Family-strengthening approaches for the prevention of youth problem behaviors. *American Psychologist, 58*, 457-465.

Lamborn, S. D., Dornbusch, S. M., & Steinberg, L. (1996). Ethnicity and community context as moderators of the relations between family decision making and adolescent adjustment. *Child Development, 67*, 283-301.

Lammers, C., Ireland, M., Resnick, M., & Blum, R. (1999). Influences on adolescents' decision to postpone onset of sexual intercourse: A survival analysis of virginity among youths aged 13 to 18 years. *Journal of Adolescent Health, 26*, 42-48.

Landrine, H., & Klonoff, E. A. (1996). The Schedule of Racist Events: A measure of racial discrimination and a study of its negative physical and mental health consequences. *Journal of Black Psychology, 22*, 144-168.

Lee, N. K., & Oei, T. P. S. (1993). The importance of alcohol expectancies and drinking refusal self-efficacy in the quantity and frequency of alcohol consumption. *Journal of Substance Abuse, 5*(4), 379-390.

Leigh, B. X., & Stall, R. (1993). Substance use and risky sexual behaviors for exposure to HIV: Issues in methodology, interpretation, and prevention. *American Psychologist, 48*, 1035-1045.

Leonard, N. R., Lester, P., Rotheram-Borus, M. J., Mattes, K., Gwadz, M., & Ferns, B. (2003). Successful recruitment and retention of participants in longitudinal behavioral research. *AIDS Education and Prevention, 15*(3), 269-281.

Lochman, J. E. (2000). Parent and family skills training in targeted prevention programs for at-risk youth. *Journal of Primary Prevention, 21*, 253-265.

Lochman, J. E., & Steenhoven, A. v. d. (2002). Family-based approaches to substance abuse prevention. *The Journal of Primary Prevention, 23*, 49-114.

Macalino, G. E., Celentano, D. D., Latkin, C., Strathdee, S. A., & Vlahov, D. (2002). Risk behaviors by audio computer-assisted self-interviews among HIV-seropositive and HIV-seronegative injection drug users. *AIDS Education & Prevention, 14*, 367-378.

MacKinnon DP, Lockwood CM, Hoffman JM, West SG, Sheets V. A comparison of methods to test mediation and other intervening variable effects. *Psychol Methods* 2002; **7**: 83-104.

Maddox, S. J., & Prinz, R. J. (2003). School bonding in children and adolescents: Conceptualization, assessment, and associated variables. *Clinical Child and Family Psychology Review, 6*, 31-49.

McKay, M. M., chasse, K. T., Paikoff, R., McKinney, L. D., Baptiste, D., Coleman, D., et al. (2004). Family-level impact of the CHAMP family program: A community collaborative effort to support urban families and reduce youth HIV risk exposure. *Family Process, 43*, 79-93.

McLaughlin, D. K., & Sachs, C. (1988). Poverty in female headed households: Residential differences. *Rural Sociology, 53*, 287-306.

Milhausen, R. R., Crosby, R., Yarber, W. L., DiClemente, R. L., Wingood, G. M., & Ding, K. (2003). Rural and nonrural African American high school students and STD/HIV sexual-risk behaviors. *American Journal of Health Behavior, 27*(4), 373.

Miller, K. S., Boyer, C. B., & Cotton, G. (2004). The STD and HIV epidemics in African American youth: Reconceptualizing approaches to risk reduction. *Journal of Black Psychology, 30*, 124-137.

Miller, K. S., Forehand, R., & Kotchick, B. A. (1999). Adolescent sexual behavior in two ethnic minority samples: The role of family variables. *Journal of Marriage and the Family, 61*, 85-98.

Miller, K. S., Forehand, R., & Kotchick, B. A. (2000). Adolescent sexual behavior in two ethnic minority groups: A multisystem perspective. *Adolescence, 35*(138), 313.

Miller, W. R., & Brown, J. M. (1991). Self-regulation as a conceptual basis for the prevention and treatment of addictive behaviours. In N. Heather, W. R. Miller & J. Greeley (Eds.), *Self-control and the addictive behaviours* (pp. 3-79). Sydney: Maxwell Macmillan Publishing.

Moffitt, T. E., Caspi, A., Dickson, N., Silva, P., & Stanton, W. (1996). Childhood-onset versus adolescent-onset antisocial conduct problems in males: Natural history from ages 3 to 18 years. *Development and Psychopathology, 8*, 399-424.

Moffitt, T. E., Caspi, A., Harrington, H., & Milne, B. J. (2002). Males on the life-course-persistent and adolescence-limited antisocial pathways: Follow-up at age 26 years. *Development and Psychopathology, 14*, 179-207.

Mrazek, P., & Haggerty, R. (Eds.). (1994). *Reducing risks for mental disorders: Frontiers for preventative intervention research*. Washington, DC: National Academy Press.

Murry, V. M. (2000). Extraordinary challenges and ordinary life experiences of Black American families. In P. C. McKenry & P. S. H (Eds.), *Family Stress and Change*. Thousand Oaks, CA: Sage Publications.

Murry, V. M., & Brody, G. H. (1999). Self-regulation and self-worth of Black children reared in economically stressed, rural, single mother-headed families: The contribution of risk and protective factors. *Journal of Family Issues, 20*, 456-482.

Murry, V. M., & Brody, G. H. (2002). Racial socialization processes in single-mother families: Linking maternal racial identity, parenting, and racial socialization in rural, single-mother families with child self-worth and self-regulation. In H. P. McAdoo (Ed.), *Black children: Social, educational, and parental environments* (pp. 97-115). Thousand Oaks, CA: Sage.

Murry, V. M., & Brody, G. H. (2004). Partnering with community stakeholders: Engaging rural African American families in basic research and the Strong African American Families preventive intervention program. *Journal of Marital and Family Therapy, 30*, 271-283.

Murry, V. M., Brody, G. H., McNair, L. D., Luo, Z., Gibbons, F. X., Gerrard, M., et al. (2005). Parental involvement promotes rural African American youths' self-pride and sexual self-concepts. *Journal of Marriage & the Family, 67*, 627-642.

Murry, V. M., Brown, P. A., Brody, G. H., Cutrona, C. E., & Simons, R. L. (2001). Racial discrimination as a moderator of the links among stress, maternal psychological functioning, and family relationships. *Journal of Marriage & the Family, 63*, 915-926.

Muthén, B., & Curran, P. (1997). General growth modeling in experimental designs: A latent variable framework for analysis and power estimation. *Psychological Methods, 2,* 371-402.

Muthén, B. O., & Muthén, L. K. (2004). *Mplus Version 3.1.* Los Angeles, CA: Authors.

National Institute of Mental Health. (1994). *The prevention of mental disorders: A national research agenda*. Washington, DC: NIMH Prevention Research Steering Committee.

National Institute On Drug Abuse. (2003). *Drug use among racial/ethnic minorities* (No. NIH Publication No. 03-03888). Washington D.C.: US Department of Health and Human Services.

Newcomb, A. F., & Bagwell, C. L. (1996). The developmental significance of children's friendship relations. In W. M. Bukowski, A. F. Newcomb & W. W. Hartup (Eds.), *The company they keep: Friendship in childhood and adolescence* (pp. 289-321). New York: Cambridge University Press.

Newcomb, M. D. (1992). Understanding the multidimensional nature of drug use and abuse: The role of consumption, risk factors, and protective factors. In M. D. Glantz & R. W. Pickens (Eds.), *Vulnerability to drug abuse* (pp. 255-297): American Psychological Association.

Nobles, W. W., Goddard, L. L., Cavil, W. E., & George, P. Y. (1987). *African-American families: Issues, insights and directions*. Oakland, CA: Black Family Institute.

Nye, C. L., Zucker, R. A., & Fitzgerald, H. E. (1995). Early intervention in the path to alcohol problems through conduct problems: Treatment involvement and child behavior change. *Journal of Consulting & Clinical Psychology, 63*, 831-840.

Oei, T. P. S., & Burrow, T. (2000). Alcohol expectancy and drinking refusal self-efficacy: A test of specificity theory. *Addictive Behaviors, 25*(4), 499-507.

Olsen, M. K, & Schafer, J., L. (2001). A two-part random effects model for semicontinuous longitudinal data. Journal of the American Statistical Association, 96, 730-745.

Oyserman, D., & Harrison, K. (1998). Implications of cultural context: African American identity and possible selves. In J. K. Swim & C. Stangor (Eds.), *Prejudice: The target's perspective* (pp. 281-300). San Diego, CA: Academic Press.

Patterson, G. R., & Yoerger, K. (1997). A developmental model for late-onset delinquency. In D. W. Osgood (Ed.), *Motivation and delinquency: Nebraska Symposium on Motivation* (Vol. 44, pp. 119-177). Lincoln: University of Nebraska Press.

Perrino, T., Gonzalez-Soldevilla, A., Pantin, H., & Szapocznik, J. (2000). The role of families in adolescent HIV prevention: A review. *Clinical Child & Family Psychology Review, 3*(2), 81-96.

Piersma, H. L., Boes, J. L., & Reaume, W. M. (1994). The Brief Symptom Inventory as an outcome measure for adolescent psychiatric inpatients. *Assessment, 1*, 151-158.

Prinz, R. J., Foster, S., Kent, R. N., & O'Leary, K. D. (1979). Multivariate assessment of conflict in distressed and nondistressed mother-adolescent dyads. *Journal of Applied Behavior Analysis, 12*, 691-700.

Proctor, B. D., & Dalaker, J. (2003). *Poverty in the United States: 2002*. Washington, D.C.: US Census Bureau.

Radloff, L. S. (1977). The CES-D Scale: A self-report depression scale for research in the general population. *Applied Psychological Measurement, 1*, 385-401.

Robinette, R. L. (1991). The relationship between the Marlowe-Crowne Form C and the validity scales of the MMPI. *Journal of Clinical Psychology, 47*, 396-399.

Rodgers, K. B. (1999). Parenting process related to sexual risk-taking behaviors of adolescent males and females. *Journal of Marriage and the Family, 61*, 99-109.

Romer, D., Stanton, B., Galbraith, J., Feigelman, S., Black, M. M., & Li, X. (1999). Parental influence on adolescent sexual behavior in high-poverty settings. *Archives of Pediatrics & Adolescent Medicine, 153*(10), 1055-1062.

Romer, D., & Stanton, B. F. (2003). Feelings about risk and the epidemic diffusion of adolescent sexual behavior. *Prevention Science, 4*(1), 39-53.

Rosenberg, P. S., Biggar, R. J., & Goedert, J. J. (1994). Declining age at HIV infection in the United States (letter). *New England Journal of Medicine, 330*, 789-790.

Rosser, B. R. S., Bockting, W. O., Rugg, D. L., Robinson, B. B. E., Ross, M. W., Bauer, G. R., et al. (2002). A randomized controlled intervention trial of a sexual health approach to long-term HIV risk reduction for men who have sex with men: Effects of the intervention on unsafe sexual behavior. *AIDS Education & Prevention, 14*, 59-71.

Rutter, M., Dunn, J., Plomin, R., Simonoff, E., Pickles, A., Maughan, B., et al. (1997). Integrating nature and nurture: Implications of person-environment correlations and interactions for developmental psychopathology. *Development and Psychopathology, 9*, 335-364.

Salazar, L. F., DiClemente, R. J., Wingood, G. M., Crosby, R. A., Harrington, K., Davies, S., et al. (2004). Self-concept and adolescents' refusal of unprotected sex: A test of mediating mechanisms among African American girls. *Prevention Science, 5*(3), 137-149.

Sampson, R. J., & Lauritsen, J. L. (1994). Violent victimization and offending: Individual, situational, and community-level risk factors. In A. J. R. Roth & A. J (Eds.), *Understanding and preventing violence* (Vol. Vol. 3, pp. 1-114). Washington, DC: National Academy Press.

Sanford, M., Offord, D., McLeod, K., Boyle, M., Byrne, C., & Hall, B. (1994). Pathways into the work force: Antecedents of school and work force status. *Journal of the American Academy of Child and Adolescent Psychiatry, 33*, 1036-1046.

Semple, S. J., Patterson, T. L., & Grant, I. (2000). Psychosocial predictors of unprotected anal intercourse in a sample of HIV positive gay men who volunteer for a sexual risk reduction intervention. *AIDS Education and Prevention, 12*, 416-430.

Simons, R. L., Lin, K., Gordon, L. C., Brody, G. H., & Conger, R. D. (2002). Community differences in the association between parenting practices and child conduct problems. *Journal of Marriage & the Family, 64*, 331-345.

Simons, R. L., Stewart, E., Gordon, L. C., Conger, R. D., & Elder, G. H. J. (2002). A test of life-course explanations for stability and change in antisocial behavior from adolescence to young adulthood. *Criminology, 40*, 401-434.

Smith, E. P., & Brookins, C. C. (1997). Toward the development of an ethnic identity measure for African American youth. *Journal of Black Psychology, 23*, 358-377.

Smith, G. T., McCarthy, D. M., & Goldman, M. S. (1995). Self-reported drinking and alcohol related problems among adolescents: Dimensionality and validity over 24 months. *Journal of Studies on Alcohol, 56*, 383-394.

Sobel, M. E. (1982). Asymptotic intervals for indirect effects in structural equations models. In S. Leinhart (Ed.), *Sociological methodology* (pp. 290-312). San Francisco: Jossey-Bass.

Spoth, R., Redmond, C., Haggerty, K., & Ward, T. (1995). A controlled parenting skills outcomes study examining individual differences and attendance effects. *Journal of Marriage & the Family, 57*, 449-464.

Spoth, R., Redmond, C., & Shin, C. (1998). Direct and indirect latent-variable parenting outcomes of two universal family-focused preventive interventions: Extending a public health-oriented research base. *Journal of Consulting & Clinical Psychology, 66*, 385-399.

Spoth, R., Redmond, C., & Shin, C. (2001). Randomized trial of brief family interventions for general populations: Adolescent substance use outcomes 4 years following baseline. *Journal of Consulting & Clinical Psychology, 69*, 627-642.

Sroufe, L. A., & Fleeson, J. (1986). Attachment and the construction of relationships. In W. H. Rubin & Z (Eds.), *Relationships and development* (pp. 51-71). New York: Cambridge University Press.

St Lawrence, J. S., & Scott, C. P. (1996). Examination of the relationship between African American adolescents' condom use at sexual onset and later sexual behavior: Implications for condom distribution programs. *AIDS Education & Prevention, 8*(3), 258-266.

St. Lawrence, J. S. (1999). Emerging behavioral strategies for the prevention of HIV in rural areas. *The Journal of Rural Health, 15*, 335-343.

St. Lawrence, J. S., Reitman, D., Jefferson, K. W., & Alleyne, E. (1994). Factor structure and validation of an adolescent version of the Condom Attitude Scale: An instrument for measuring adolescents' attitudes toward condoms. *Psychological Assessment, 6*(4), 352-359.

Steele, C. M. (1997a). Race and the schooling of Black Americans. In L. A. Peplau & S. E. Taylor (Eds.), *Sociocultural perspectives in social psychology: Current readings* (pp. 359-371). Upper Saddle River, NJ: Prentice-Hall.

Steele, C. M. (1997b). A threat in the air: How stereotypes shape intellectual identity and performance. *American Psychologist, 52*, 613-629.

Stein, J. A., & Nyamathi, A. (1999). Gender differences in relationships among stress, coping, and health risk behaviors in impoverished, minority populations. *Personality & Individual Differences, 26*(1), 141-157.

Stevenson, H. C. (1997). Managing anger: Protective, proactive, or adaptive racial socialization identity profiles and manhood development. *Journal of Prevention and Intervention in the Community, 16*, 35-61.

Stevenson, H. C., Reed, J., Bodison, P., & Bishop, A. (1997). Racism stress management: Racial socialization beliefs and the experience of depression and anger in African American youth. *Youth and Society, 29*, 197-222.

Sudarkasa, N. (1988). Interpreting the African heritage in Afro-American family organization. In H. P. McAdoo (Ed.), *Black families* (pp. 27-43). Beverly Hills, CA: Sage.

Sussman, S., & Johnson, C. (1996). Drug abuse prevention: Program and research recommendations. *American Behavioral Scientist, 39*(7), 787-789.

Tapert, S. F., Aarons, G. A., Sedlar, G. R., & Brown, S. A. (2001). Adolescent substance use and sexual risk-taking behavior. *Journal of Adolescent Health, 28*, 181-189.

Tickamyer, A. R., & Duncan, C. M. (1990). Poverty and opportunity structure in rural America. *Annual Review of Sociology, 16*, 67-86.

Tobler, N. S., Roona, M. R., Ochshorn, P., Marshall, D. G., Streke, A. V., & Stackpole, K. M. (2000). School-based adolescent drug prevention programs: 1998 meta-analysis. *Journal of Primary Prevention, 20*(4), 275-336.

Tucker, J. S., Orlando, M., & Ellickson, P. L. (2003). Patterns and correlates of binge drinking trajectories from early adolescence to young adulthood. *Health Psychology, 22*, 79-87.

Wagenfeld, M. O., Murray, J. D., Mohatt, D. F., & DeBruyn, J. C. (1994). *Mental Health and Rural America: 1980-1993; An Overview and Annotated Bibliography* (No. NIH Pub. No. 94-3500). Washington, DC:: Office of Rural Health, Health Resources and Services Administration; Office of Rural Mental Health Research, National Institute of Mental Health, National Institutes of Health.

Walker, S. S. C., & DeLone, M. (2000). *The color of justice: Race, ethnicity, and crime in America*. Belmont, CA: Wadsworth.

Wallace, J. M. J. (1999). The social ecology of addiction: Race, risk, and resilience. *Pediatrics, 103*, 1122-1127.

Wallace, J. M. J. (2002). Preventing substance abuse among African American children and youth: Race differences in risk exposure and vulnerability. *The Journal of Primary Prevention, 22*, 235-261.

Wallace, J. M. J., & Bachman, J. G. (1991). Explaining racial/ethnic differences in adolescent drug use: The impact of background and lifestyle. *Social Problems, 38*, 333-357.

Wallace, J. M. J., Bachman, J. G., O'Malley, P. M., Johnston, L. D., Schulenberg, J. E., & Cooper, S. M. (2002). Tobacco, alcohol, and illicit drug use: Racial and ethnic differences among U.S. high school seniors, 1976-2000. *Public health reports, 117 Suppl 1*, S67-75.

Wang, C.-P., Brown, C. H., & Bandeen-Roche, K. (1995). Model diagnostics for general growth mixture models: Examining the impact of a preventive intervention on differential pathways to aggressive behavior. *Journal of the American Statistical Association, 100*, 1054-1076.

Whitaker, D. J., & Miller, K. S. (2000). Parent-adolescent discussions about sex and condoms: Impact on peer influences of sexual risk behavior. *Journal of Adolescent Research, 15*(2), 251-273.

White, H. R., & Labouvie, E. W. (1989). Towards the assessment of adolescent problem drinking. *Journal of Studies on Alcohol, 50*, 30-37.

White, H. R., Pandina, R. J., & Chen, P.-H. (2002). Developmental trajectories of cigarette use from early adolescence into young adulthood. *Drug and Alcohol Dependence, 65*, 167-178.

Williams, C. L., Toomey, T. L., McGovern, P., & Perry, A. C. W. L. (1995). Development, reliability, and validity of self-report alcohol-use measures with young adolescents. *Journal of Child and Adolescent Substance Abuse, 4*, 17-40.

Willis, W. (1992). Families with African American roots. In E. W. Lynch & M. J. Hansons (Eds.), *Developing cross-cultural competence: A guide for working with young children and their families* (pp. 121-150). Baltimore: Brookes.

Wills, T. A., Blechman, E. A., & McNamara, G. (1996). Family support, coping, and competence. In E. M. B. E. A. Hetherington (Ed.), *Stress, coping, and resiliency in children and families.* (pp. 107-133). Hillsdale, NJ, England: Lawrence Erlbaum Associates, Inc.

Wills, T. A., Gibbons, F. X., Gerrard, M., & Brody, G. H. (2000). Protection and vulnerability processes relevant for early onset of substance use: A test among African American children. *Health Psychology, 19*(3), 253-263.

Wills, T. A., Gibbons, F. X., Gerrard, M., Murry, V. M., & Brody, G. H. (2003). Family communication and religiosity related to substance use and sexual behavior in early adolescence: A test for pathways through self-control and prototype perceptions. *Psychology of Addictive Behaviors, 17*, 312-323.

Wills, T. A., & Hirky, A. E. (1996). Coping and substance abuse: A theoretical model and review of the evidence. In M. E. N. S. Zeidner (Ed.), *Handbook of coping: Theory, research, applications.* (pp. 279-302). Oxford, England: John Wiley & Sons.

Wills, T. A., Sandy, J. M., & Yaeger, A. M. (2001). Time perspective and early-onset substance use: A model based on stress-coping theory. *Psychology of Addictive Behaviors, 15*(2), 118-125.

Wills, T. A., Sandy, J. M., & Yaeger, A. M. (2002). Moderators of the relation between substance use level and problems: Test of a self-regulation model in middle adolescence. *Journal of Abnormal Psychology, 111*(1), 3-21.

Wills, T. A., & Shiffman, S. (1985). Coping and substance abuse: A conceptual framework. In S. Shiffman & T. A. Wills (Eds.), *Coping and substance abuse* (pp. 1-21). New York: Academic Press.

Wills, T. A., & Shinar, O. (2000). Measuring perceived and received social support. In S. Cohen & L. G. Underwood (Eds.), *Social support measurement and intervention: A guide for health and social scientists* (pp. 86-135). London: Oxford University Press.

Wills, T. A., & Stoolmiller, M. (2002). The role of self-control in early escalation of substance use: A time-varying analysis. *Journal of Consulting & Clinical Psychology, 70*(4), 986-997.

Wills, T. A., Vaccaro, D., & McNamara, G. (1992). The role of life events, family support, and competence in adolescent substance use: a test of vulnerability and protective factors. *American journal of community psychology Am J Community Psychol, 20*(3), 349-374.

Wills, T. A., Windle, M., & Cleary, S. D. (1998). Temperament and novelty seeking in adolescent substance use: Convergence of dimensions of temperament with constructs from Cloninger's theory. *Journal of Personality and Social Psychology, 74*, 387-406.

Wills, T. A., Yaeger, A. M., & Sandy, J. M. (2003). Buffering effect of religiosity for adolescent substance use. *Psychology of Addictive Behaviors, 17*(1), 24-31.

Wingood, G. M., & DiClemente, R. J. (1998a). Gender-related correlates and predictors of consistent condom use among young adult African-American women: A prospective analysis. *International Journal of STD & AIDS, 9*(3), 139-145.

Wingood, G. M., & DiClemente, R. J. (1998b). The influence of psychosocial factors, alcohol, drug use on African-American women's high-risk sexual behavior. *American Journal of Preventive Medicine, 15*(1), 54-59.

Wong, C. A., Eccles, J. S., & Sameroff, A. (2003). The influence of ethnic discrimination and ethnic identification on African American adolescents' school and socioemotional adjustment. *Journal of Personality, 71*, 1197-1232.

Young, R. M., & Oei, T. P. S. (2000). The predictive utility of drinking refusal self-efficacy and alcohol expectancy: A diary-based study of tension reduction. *Addictive Behaviors, 25*(3), 415-421.

Young, R. S., Oei, T. P., & Crook, C. M. (1991). Development of a drinking self efficacy scale. *Journal of Psychopathology and Behavioral Assessment, 13*, 1-15.

Zimbardo, P. G., & Boyd, J. N. (1999). Putting time in perspective: A valid, reliable individual-differences metric. *Journal of Personality & Social Psychology, 77*, 1271-1288.

Zimbardo, P. G., Keough, K. A., & Boyd, J. N. (1997). Present time perspective as a predictor of risky driving. *Personality and Individual Differences, 23*, 1007-1023.

Zimmerman, M. A., & Schmeelk-Cone, K. H. (2003). A longitudinal analysis of adolescent substance use and school motivation among African American youth. *Journal of Research on Adolescence, 13*, 185-210.

Zucker, R. A. (1994). Pathways to alcohol problems and alcoholism: A developmental account of the evidence for multiple alcoholisms and for contextual contributions to risk. In R. A. Zucker, T. Howard & G. M. Boyd (Eds.), *The development of alcohol problems: Exploring the biopsychosocial matrix of risk* (pp. 255-290). Rockville, MD: National Instituted on Alcohol Abuse and Alcoholism.

Zucker, R. A., Chermack, S. T., & Curran, G. M. (2000). Alcoholism: A life span perspective on etiology and course. In A. J. Sameroff & M. Lewis (Eds.), *Handbook of developmental psychopathology (2nd ed.).* (pp. 569-587): Kluwer Academic Publishers.

H. CONSORTIUM/CONTRACTUAL ARRANGEMENTS: Not applicable.

J. CONSULTANTS

Dr. C. Hendricks Brown, Professor of Epidemiology and Biostatistics at the University of South Florida, will consult with the investigators on data analysis. He has expertise in methods of statistical analysis for preventive intervention field trials

AIM

A. SPECIFIC AIMS

Our sample will consist of 370 families with a high school senior, half of whom will be assigned randomly to a prevention group (SAAF—EAP) and half of whom will be assigned to a control group. Pre-intervention, post-intervention, and follow-up assessments of youths' substance use and secondary outcomes will be conducted with the entire sample. Our specific aims are as follows:

1. To test the hypothesis that rural African American emerging adults randomly assigned to participate in SAAF—EAP, compared to control participants, will demonstrate lower rates of substance use initiation, and that those who already use substances (e.g., cigarettes; alcohol, including binge drinking; marijuana and other illicit substances) will use them less frequently and consume lower quantities, at each follow-up assessment.
2. To test the hypothesis that SAAF—EAP primary caregivers and emerging adults, compared to control participants, will report higher levels of autonomy-promoting parenting and responsiveness in family relationships at each follow-up assessment.

D. RESEARCH METHODS

D1. Participants

A random sample of 370 rural African American families with a son or daughter in the last semester of public high school from each of 6 counties in central Georgia will participate in the study.

D1.1. Random Assignment, Other Programs, and Contamination: Families will be assigned randomly to either the prevention or control condition.

D1.2. Inclusion/Exclusion criteria: We will recruit all youth from public schools in their senior year of high school in the target counties that self-report being African American or Black and age 17-19. We will exclude from the pool of potential participants a small number of students whose participation in the intervention's group-based activities may create negative iatrogenic effects (Dishion, McCord, & Poulin, 1999). Students who have been suspended from school more than once and those who were expelled from a high school other than the one they are attending will be disqualified.

D1.3. Family Recruitment and Retention Strategies: The recruitment process will be carried out in the following sequence: (1) a letter will be mailed to parents/guardians from the school principal informing them of the study, (2) a home visit will be made by a community liaison to obtain active consent from primary caregiver and student to implement the screening procedures described previously, and (3) families will be invited to participate with active consent obtained from primary caregivers and active assent from emerging adults.

D1.3a. Principals' letter: Each primary caregiver of an African American high school senior who is selected randomly from the pool of potential participants will receive a letter from the school principal describing the study and the collaboration with the University of Georgia (UGA). Caregivers will be informed that they will be contacted by representatives associated with UGA (community liaisons) and they and their high school seniors may be invited to participate.

D1.3b. Community liaisons' home visits. Community liaisons will schedule initial home visits to families of students selected randomly from the potential participant pool. During this visit, the liaison will provide the family and student with information about the study and tell them that, based on school information, it is possible that they may be invited to participate. The community liaison will give consent/assent forms to the primary caregiver and student. A student will be classified as refusing at this point if (a) the caregiver or student does not wish to take part in the study or (b) the caregiver or student does not consent to allow UGA researchers to obtain school information. These students will be removed from the sample pool and will not be screened for the exclusionary criteria.

D1.3c. Monetary incentives: At each pretest, posttest, and follow-up assessment in the proposed project, emerging adults will receive $50 and participating caregivers will receive $100.

D1.3d. Retention. Following each home visit and prevention session, community liaisons contact participants to inquire about the quality of the experience and to answer questions. Between data collections, community liaisons call families on a regular basis. Community liaisons will obtain contact information for emerging adults who leave their parents' homes and call these young people directly at their own residences. In the event that a phone line is temporarily disconnected, community liaisons visit the families' homes or the residences of emerging adults who live apart from their families. The families also provide us with contact information for three individuals who will always know their whereabouts; these lists are updated at each home

visit. All emerging adults and their families will receive a semiannual newsletter that will include a stamped address correction form to facilitate the updating of address lists before post office forwarding orders expire.

D2. Overview of the Prevention and Research Procedures

All prevention and control families will participate in a pretest, a posttest, and a follow-up assessment that takes place 9 months after the posttest. Prevention families will participate in a 6-week intervention while control families receive, via postal mail, information about emerging adult development, stress, diet, and exercise. The posttest data collection will take place 3 months after prevention training concludes to allow any transitory immediate effects to dissipate. Table 3 presents the time line for all activities included in the scope of management, this proposal. If we are able to obtain additional funding later, we will continue to conduct booster sessions and follow-up studies with the families as the youths progress through emerging adulthood.

Table 3. Time line.

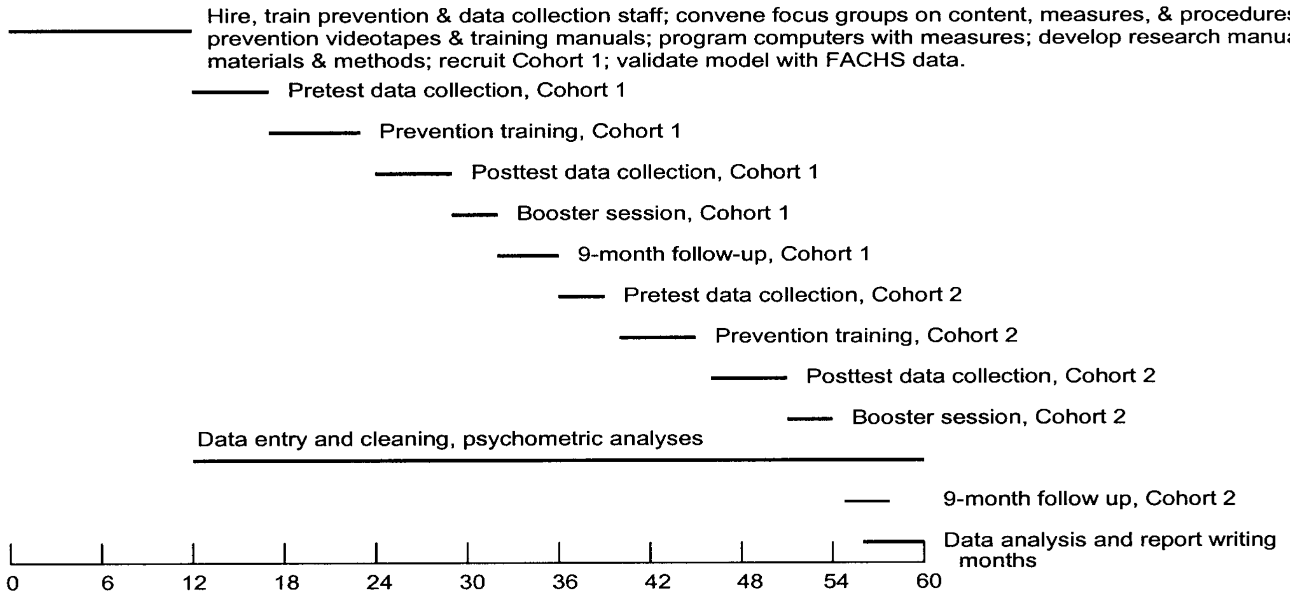

D3. Prevention Development Plan

D3.1. Development of the Prevention Program with the Assistance of Community Members: Project staff will meet with two groups of 10 adults and two groups of 10 youths who are representative of the African American communities in the participating counties to discuss the prevention curriculum modules and the proposed training process. The focus group members will be asked for feedback about the propriety of the curriculum for rural African American families and emerging adults; the curriculum will be revised accordingly. The focus group will evaluate the revised curriculum to insure that the changes effectively addressed their suggestions.

A pilot intervention feasibility study will be conducted with a sample of 10 African American emerging adults and their parents. The pilot will be videotaped and a staff member from the Center will view the tapes and take notes on each session. Session logistics, as well as the relevance and appropriateness of activities and materials, will be assessed and any necessary revisions will be made to the final curriculum.

D3.2. Implementation of the Prevention Program: Procedures for recruiting and training the group leaders, insuring fidelity to the prevention curriculum, and engaging participating families will follow the same guidelines that Brody and Murry used successfully in SAAF (Brody et al, in press; Murry & Brody, in press).

D3.2a. Group leaders: Three-person teams will lead prevention groups, each of which will include ~10 families. All group leaders will be African Americans. The leaders will receive 30 hours of training at the Center for Family Research of the University of Georgia. The training will address content delivery within a structured group process format, implementation of specific curriculum activities, guided practice in delivering and pacing curriculum segments, and leader self-care. Drs. Murry, Brody, Arnett, Brown, and Smith, along with Prevention Coordinator Tracy Anderson, will prepare a leaders' guide. Didactic material, role-playing exercises, and modeling will be used to teach the protocol for each session. Prior to completion of training, group leaders must demonstrate mastery of the prevention programming by scoring 100% on a written examination covering

the prevention material. Leaders will meet with Ms. Anderson for 2 hours each week to review the previous week's sessions and prepare for those in the coming week.

D3.2b. *Treatment integrity:* Standardized procedures will be followed to insure the prevention sessions' integrity. The intervention manual will include a detailed format to be used for each session. Each team will be provided with materials designed to facilitate correct execution of the session protocol, including an outline of each session, a checklist of the materials necessary for each activity, the specific theme of each task, and forms for in-session notes. Each session's content and instruction process will be documented on videotape. Leaders will receive ongoing supervision throughout the study, including unannounced direct observations by the field supervisor. To assess fidelity to the prevention program, videotapes of all sessions will be scored for coverage of the curriculum and adherence to the protocol. As in SAAF, fidelity instruments will be developed to score the caregiver, emerging adult, and family sessions. These strategies have been found to increase consistency among group leaders (Nye et al., 1995). Presentation of targeted processes on videotapes also will help to ensure the intervention's integrity.

D3.2c. *Logistics:* In each of the prevention counties, two meeting sites will be established at community facilities in central locations that are easily accessible to program participants. Local churches and schools provide ample space for classrooms, waiting areas, and parking.

D3.2d. *Engagement procedures:* Several strategies used in SAAF to address scheduling conflicts, transportation needs, and childcare requirements will be used in the proposed program. During the pretest data collection, family members will be asked about their weekly schedules and sessions will be planned to accommodate as many families as possible. During the first half hour of each meeting, dinner will be served to family members attending the prevention sessions. Leaders will arrange transportation for any families who need it and child care will be provided for any children who come with the families. An intensive reminder protocol also will be used throughout the course of the program. Community liaisons will call all participants before the program begins and prior to each weekly prevention session to encourage their involvement. Reminder postcards will be sent from the Center each week, highlighting the focus of the upcoming meeting. A community liaison will call any participants who miss a meeting to remind them of the next meeting and address any barriers to attendance.

D3.3. *Program Structure:* The delivery system used in the SAAF program will be adapted and used to structure the proposed intervention. The prevention program will consist of 6 consecutive weekly meetings, with separate family member and emerging adult skill-building curricula and a family curriculum. Each meeting will include separate, concurrent sessions for family members and emerging adults, followed by joint sessions during which the emerging adults and their family members review, discuss, and practice the skills they learned in their separate sessions. Videotapes portraying family interactions and intrapersonal processes will be used in the emerging adult, family member, and joint family sessions to ensure consistent presentation of key points. Each session will include a homework review, didactic presentation of information, group discussion, and skill-building activities that reinforce learning. The concurrent and family sessions will each last 1 hour; thus, emerging adults and family members will receive 12 hours of prevention training. During the same weeks that the intervention families participate in the prevention sessions, families in the control group will receive weekly leaflets via postal mail. They will describe emerging adult social, emotional, and identity development; stress management; and information about diet and exercise needs.

D3.4. *Prevention Program:* The content of the program is based on the causative model that we developed based on Arnett's and on Brody and Murry's research programs. The prevention curriculum objectives for each session can be found in Appendix 6.

D3.4a. *Autonomy-promoting and responsive family relationships:* The caregivers' curriculum will focus on providing emerging adults with developmentally appropriate instrumental and emotional support; providing clear expectations for emerging adults' behavior; engaging emerging adults in discussions about future plans for meeting expectations and responsibilities; fostering racial pride; conveying adaptive strategies for coping with discrimination; modeling and encouraging nonavoidant ways of coping with various life stresses; developing strategies for maintaining affectively close and involved relationships while promoting autonomy; and articulating negative expectations for substance use.

D3.4b. *Self-control, future orientation, and emotion control*: The prevention topics for emerging adults will address setting goals and developing courses of action to meet them; using time productively and keeping commitments; identifying coping strategies to use when confronting financial, family, friendship, or

discrimination-related stress; applying nonavoidant, problem-focused coping strategies to various stressful situations; forming educational and occupational goals with step-by-step plans for attaining them; and dealing with institutional racism.

D3.4c. Substance use prototypes and images: The prevention topics for emerging adults include distinguishing unintentional from intentional behavior; understanding the prototypes or images that they hold of substance-using agemates; learning that most of their peers hold more negative than positive images of substance-using agemates; recognizing risk-conducive situations, forming plans to avoid risks; and considering actions they can take if they find themselves in high-risk situations.

D4. Data Collection

One 2-hour home visit will be made to each family for data collection at the pretest, posttest, and follow-up assessments that take place 9 months after the posttest. To minimize cultural bias, African American students and community members will serve as home visitors.

D4.1. Intervention Assessment: All self-report data from emerging adults and their parents will be gathered during private interviews with a researcher, with no other family members present or able to overhear the conversation. At no time during the presentation of the self-report instruments will the researchers assume that a family member can read; this literacy concern is one of the reasons for presenting the questionnaires in an interview format. The interviews will be conducted using Computer Assisted Interviewing (CAI) technology with laptop computers. One item at a time will be displayed on the computer screen; the interviewer will read each item to the participant and enter the answer into the computer. The use of CAI creates an easy interview pace; reduces missing data due to skipped questions, out-of-range responses and inconsistent answers; and increases administration consistency.

D4.2. Process Evaluation of the Prevention Program: We will use the number of program sessions attended to measure intervention exposure. Using session attendance and program fidelity data (see Section 3.2b), a dosage score will be calculated for each study participant. Both parents in two-parent families will be encouraged to attend each session of the program; in single-parent families, the parent and an extended family member who assisted with the emerging adult's upbringing will be encouraged to attend. If two adults cannot come, attendance of one will be encouraged as preferable to attendance of neither. At the end of each prevention session, the leaders will rate group responsiveness, engagement, and supportiveness using 5-point process evaluation scales.

D5. Measures

We will use multimethod, multiple indicator strategies to assess the constructs underlying the intervention theory.

D5.1. Demographics: The demographic interview was designed and tested in Georgia with samples composed of single mothers. Primary caregivers will report their yearly income, per capita income, employment status, duration of unemployment during the past 2 years, hours worked per week, education level, numbers of children and adults in the household, and relations of all household members to one another.

D5.2. Autonomy-Promoting and Responsive Family Relationships: Four characteristics of family relationships are hypothesized to prevent substance use: (1) emotional and instrumental support, (2) affectively positive relationships featuring open communication, (3) adaptive racial socialization, and (4) setting expectations for emerging adults and providing support for meeting them.

D5.2a. Emotional and instrumental support: Emerging adults and parents will complete the Family Support Inventory (FSI; Wills, Vaccaro, & McNamara, 1992), a14-item measure of perceived availability of emotional and instrumental support from family members. Internal consistency ranged from .82 to .88 for emotional support and .76 to .83 for instrumental support with multiethnic samples of adolescents. The scales have good psychometric properties (Wills & Shinar, 2000) and consistently predicted less substance use, greater self-regulation, and more resilient coping in multiethnic samples of adolescents (Wills, Blechman, & McNamara, 1996; Wills, & Cleary, 1996; Wills, Windle & Cleary, 1998). In addition to the FSI, four items that Carver, Scheier, and Weintraub (1989) developed will be used to assess the frequency with which family members provide emotional support. These items were modified slightly in response to focus groups' feedback. The items' Cronbach's alphas in our prior prevention research ranged from .78 to .87 (Brody et al., in press); with multiethnic samples, they predicted more self-regulated coping and less adolescent substance use (Wills, Resko, Ainette, & Mendoza, in press).

D5.2b. Affectively positive relationships featuring open communication: Parents and emerging adults

will complete the Interaction Behavior Questionnaire (IBQ; Prinz, Foster, Kent, & O'Leary, 1979), which assesses affective involvement and positivity in family relationships. Cronbach's alphas for this scale with one of Brody and Murry's longitudinal research samples (Brody et al., 2002) were .85 for adult caregivers and .79 for youths.

The Discussion Quality Scale (Brody et al., 1998) will be used to assess family discussions that involve emerging adults. Parents and emerging adults will be asked about the frequency and bi-directionality of their discussions regarding school, religion, and peers. Cronbach's alphas were .84 for mothers and .79 for youths. In the proposed study, four topics relevant to emerging adults and intervention-targeted behavior will be added: future work or education, substance use, management of racial discrimination in adult contexts, and plans for the future. Focus groups will evaluate these items prior to their addition to the assessment protocol.

Conflictual and ineffective communication will be assessed from parents' and emerging adults' perspectives using a modified version of the Ineffective Arguing Inventory (Kurdek, 1994). We adapted this instrument for our work with rural African American mothers and youths (Brody et al., 2002, 2004). The items' wording was revised based on focus groups' feedback and one item was dropped due to poor reliability. Internal consistencies for the resulting 7-item scale were .78 for parents and .70 for youths participating in Brody and Murry's longitudinal research.

D5.2c. *Adaptive racial socialization*: Emerging adults and parents will complete Stevenson's (1997) Scale of Racial Socialization for Adolescents (SORS-A). The measure includes 4 subscales that assess various aspects of adaptive racial socialization. Cultural Pride Reinforcement indexes encouragement to take pride in African American culture; Racism Awareness Teaching involves helping emerging adults to be alert to racism and its consequences; Life Achievement Struggling emphasizes overcoming obstacles to achievement; and Extended Family Caring measures relatives' caregiving support. Brody and Murry reported good reliability and construct validity for this measure, which evinced sensitivity to prevention programming effects in SAAF (Brody et al., in press; Murry & Brody, in press.) For the present study, we developed additional items that focus specifically on skills for managing racism in emerging adults' environments. Focus groups of rural African American parents will review the new items before the items are added to the assessment protocol.

D5.2d. *Setting expectations and responsibilities for emerging adults:* Emerging adults and parents will report the extent to which they discuss the future (Eccles & Barber, 1993), using items such as, "How much do you talk to your child about the job they would like to have when they're grown up?" and "My mom is helpful with plans for future education." McCabe and Barnett (2000) obtained Cronbach's alphas of .92 for youth reports and .90 for parent reports with a sample of African American families.

D5.3. *Additional Individual and Family Risk Factors:*

D5.3a. *Parenting congruence*: Parents will complete Ahrons' (1981) Quality of Coparenting Scales—Revised, which assess agreement on parenting issues. Cronbach's alpha from Brody and Murry's research with a rural Georgia sample was .79 (Brody et al., 2002).

D5.3b. *Parental conflict*: Parents will complete the O'Leary-Porter Scale (Porter & O'Leary, 1980), indicating frequency of conflict with one another in the emerging adult's presence. Cronbach's alpha from our previous research with a rural Georgia sample was .79 (Brody et al., 2002).

D5.3c. *Parental depression*: The Center for Epidemiologic Studies Depression Scale (Radloff, 1977), which was designed for use with community samples, will be used to assess depression among parents. With rural Georgia samples, internal consistency averaged .84 and the scale was associated with competence-promoting parenting practices and co-caregiver conflict (see Section C).

D5.3d. *Parental substance use*: Parents and will respond to the Parent Alcohol/Drug Use History (Collins, Parks, & Marlatt, 1985). Items assess drinking in a typical week and both lifetime and recent use of marijuana, stimulants, sedatives, psychedelic drugs, cocaine, heroin, and opiates. Internal consistencies for caregivers in our prior prevention research ranged from .79 to .81.

D5.3e. Emerging adults' psychological functioning. Emerging adults' parents will complete the Child Behavior Checklist (Achenbach, 2000).

D5.4. *Community/Social Contextual Characteristics:*

D5.4a. *Discrimination*: Emerging adults and parents will report their experiences with racism using the Schedule of Racist Events (SRE; Landrine & Klonoff, 1996) and the Racist Hassles Questionnaire. We will use the Frequency Of Unfair Treatment Based On Race and Stress Of Racial Unfairness subscales of the SRE. Respondents rate the occurrence and stressfulness of racially based unfair treatment by school officials,

business employees, legal system officials, neighbors, church members, friends, and strangers. In Brody and Murry's developmental research, Cronbach's alphas for youths and caregivers exceeded .75 for both subscales.

D5.4b. *Restricted educational and occupational opportunities:* The Total Resources subscale (α = .95) of the Community Problems and Resources Scale (CPRS; Forehand & Brody, 2000), which we have used previously in our longitudinal studies, includes 19 positive community characteristics such as job training, educational facilities, social organizations, and transportation. For the proposed study, additional items were developed to target specific educational and job opportunities. Focus groups will evaluate these items before the items are added to the assessment.

D5.4c. *Financial stress:* Chronic financial difficulties will be assessed using scales that Conger and associates (e.g., Conger & Elder, 1994) developed as part of their work with rural families and subsequently used with the FACHS sample: Unmet Material Needs, Can't Make Ends Meet, Financial Adjustments, and Negative Financial Events. Reliabilities for the FACHS sample ranged from .69 to .80.

D5.4d. *Personal life stressors:* The number of negative life events that parents and emerging adults experienced in the past 12 months will be measured using a checklist that includes events such as criminal victimization, serious illness or injury, legal problems, and marital separation or divorce. This measure has been used in past longitudinal research on rural families (Conger & Elder, 1994). When used with African American adults in FACHS, it was significantly correlated with distress and severity of financial problems (ps < .001).

D5.6. Emerging Adults' Protective Intrapersonal Processes:

D5.6a. Self-control: Emerging adults will complete the Self-Control Inventory (SCI; Wills & Stoolmiller, 2002), a 25-item instrument with subscales that measure good (soothability, dependability, planning, problem solving) and poor (impatience, distractibility, angerability) self-control. With adolescent samples, the scales' reliabilities ranged from .73 to .83, and the scores predicted substance use problems in both multiethnic (Wills et al., 2001, 2002) and African American (Wills, Gibbons, et al., 2000; Wills, Gibbons, et al., 2003) samples.

D5.6b. Emotion regulation: Emerging adults will report their coping styles using the Coping Responses Inventory (CRI; Moos, 1990,1993). The CRI has been widely used with samples of adults who use substances; the eight subscales' internal consistencies range from .61 to .74 and are intercorrelated (average r =. 29; Moos, 1997). Emerging adults will also complete Wills and Hirky's (1996) coping processes inventory. The measure provides additional, situation-specific information on emerging adults' ways of dealing with typical problems at home and school. The proposed project will include the measure's anger-based coping and helpless coping subscales. In FACHS, the subscales' Cronbach's alphas ranged from .74 to .99 for caregivers and from .62 to .88 for youths (Gibbons & Simons, 2001).

D5.6c. *Future orientation:* Future orientation will be assessed using the future, present/hedonistic, and present/fatalistic subscales of the Time Perspective Inventory (Zimbardo & Boyd, 1997). The future subscale reflects planning goals and ways to achieve them (α =. 77). The present/hedonistic subscale represents an orientation toward present enjoyment, pleasure, and excitement (α =. 79). The present/fatalistic subscale indexes a belief that the future is predestined and cannot be influenced by individuals' actions (α =. 74). Responses to an adapted version of this measure from an urban multiethnic sample were associated with self-regulation, anger-based coping, and self-esteem (Wills et al., 2001).

D5.6d. *Racial identity:* Racial identity will be assessed using the Centrality and Regard subscales of the Multidimensional Model of Racial Identity for African Americans (Sellers et al., 1997). Brody and Murry have used these scales successfully in their research (Murry et al., 2004). The Centrality subscale (α = .70) measures the extent to which being African American is central to the respondents' definitions of themselves. Regard (α = .73) refers to affective and evaluative perceptions of one's race.

D5.7. *Emerging Adults' Substance Use Prototypes and Behavioral Willingness:* A short version of Gibbons and Gerrard's (1995, 1997) risk prototype scale will be included. Participants will be given a brief explanation of the prototype concept (Gibbons & Gerrard, 1995; Gibbons, Gerrard, & Boney-McCoy, 1995), then be asked to "think for a minute about the type of person your age who (drinks alcohol; smokes pot)" and to indicate the extent to which the following terms describes that person: smart, popular, cool, dull (boring), good-looking, and childish. A parallel question assessing prototypes of non-users will be included (Gerrard et al., 1999). Participants also will indicate how similar they believe they are to each of these prototypes. Negative items will be reverse scored, so that high scores will reflect a favorable image. Cronbach's alphas in the

FACHS exceeded .70 for each scale.

Willingness to use substances will be assessed using items developed in our research with rural adolescents (Gerrard et al., 1999; Gibbons et al., 1998). Emerging adults will be asked to imagine a risk-conducive situation: "Suppose you were with some friends at a party and one of them offered you some kind of alcoholic drink." They will then be asked to indicate how likely they would be to do each of the following: leave the situation, tell them "no thanks," take it and try it, and have more than one drink. In prior studies with rural African American adolescents (Gibbons et al., in press), Cronbach's alpha for this scale was .86.

D5.8. Emerging Adults' Substance Use: The substance use assessment is designed to address the multidimensional nature of use (Newcomb, 1992). From three well-validated measures, items were selected for their sensitivity to intervention effects at several levels of substance use. At the initiation level, group differences in the proportion of participants who transition from nonuse to use during the study can be detected. At the escalation level, group differences in the proportion of participants who increase their frequency/intensity of use during the study can be assessed. At the problem use level, group differences in specific substance-related behaviors and problems can be indexed. The basic items in the measure are those included in the Monitoring the Future study (Johnston, Bachman, & O'Malley, 2002), which assess lifetime, annual, and 30-day prevalence and frequency of cigarette smoking, alcohol use, binge drinking, and marijuana use. For the purposes of the proposed study, a 6-month assessment will be added. A checklist of other drugs includes lifetime use of LSD/acid, Ecstasy, cocaine, heroin, other opiates, amphetamines, non-prescribed tranquilizers, and inhalants. Problems associated with substance use will be indexed using items from the Minnesota Student Survey for high school students (Harrison et al., 1998) assessing dependence (e.g., had to use more to get same effect, tried to cut down but couldn't) and negative consequences of use (e.g., use hurt relationships with friends or family, use interfered with other activities, use resulted in problems with law, missed school or work because of use).

D6. Plan of Analyses

D6.1. Preliminary Analyses: Prior to executing psychometric analyses, distribution kurtosis and skew, means, and variances will be examined for each continuous variable, and appropriate transformations will be conducted on indicators with extreme kurtosis or skew. For limited dependent variables (dichotomous, count, or censored variables, see below), we will use statistical techniques appropriate for each variable, including logistic regression, Poisson, negative binomial, or Tobit regression, or proportional hazard models in the case of models for time-to-event outcomes, such as age at substance use initiation.

D6.1a. Reliability, construct development, and multi-informant analyses: For more than a decade, we have used multi-informant data from rural African Americans in our analyses. Building latent constructs from multi-informant data involves several steps. First, the construct is operationally defined, with items grouped into scales conceptually as well as through factor analysis and other multidimensional scaling techniques (Bentler, 1976). Items that meet internal consistency criteria (Cronbach's alpha) are then examined in a confirmatory factor analysis with SEM to demonstrate that the construct can be distinguished from other constructs and to establish convergent and discriminant validity. Because we will use many well-validated scales, we expect instrumentation issues to pose minimal analytic difficulties.

D6.1b. Attrition and missing data issues: For the proposed research, we will use two approaches to deal with incomplete data: Full Information Maximum Likelihood (FIML; Arbuckle, 1996; Marini, Olsen, & Rubin, 1980; McArdle, 1994; Muthén, Kaplan, & Hollis, 1987) and Multiple Imputation (MI; Heeringa & Rodgers, 1998; Rubin, 1987; Schafer, 1997). Both techniques correctly estimate population parameters and standard errors when data are "missing at random" Rubin (1987).

D6.1c. Intent-to-treat analyses: To preserve random assignment, the analyses will include all prevention families who complete the pretest, regardless of the number of prevention sessions they attend.

D6.1d. Baseline equivalence for prevention and control groups: Before testing the hypotheses, we will use hierarchical linear modeling (HLM; Bryk and Raudenbush, 1992) to conduct statistical comparisons between prevention and control group members on all study variables. Because participants will be recruited from schools, we will use HLM to examine pretest differences among youths attending different schools. School-level effects will be included in tests of the hypotheses if they account for unique variance at baseline.

D6.2. Substance Use Outcome Measures: The assessment of substance use (see Section D5.8) covers initiation, escalation, and problem use. SAAF—EAP intervention effects will be examined for use, frequency of use, and quantity used during the past 30 days, 6 months, and 1 year. We can determine lifetime use for all

substances from the history-of-use questions, identifying movement from nonuse to initiation of use for specific substances. We will use discrete event-history models to examine SAAF-EAP effects on initiation over the 3 repeated measures.

D6.3. _Power Analysis:_ As mentioned in Section D1, 690 families will be recruited to allow for an attrition rate of 15%. Power analyses were based on an effect size of .30 (Durlak & Wells, in press). Table 9.4.1 in Cohen (1977) can be used to calculate a necessary sample size of around 150 families per group to detect differences between the prevention and comparison groups with an alpha of .01 and a power of .90 for the dependent measures. For the analyses presented below using structural equation modeling (SEM), we considered both sample size and statistical power issues. The literature on sample size for latent variable structural equation analysis suggests that 150 cases are enough to ensure a reliable estimation of the model (see discussion by Tanaka, 1987). Much of this literature, however, is based on Monte Carlo studies of factor analysis models involving relatively few parameters (e.g., Boomsma, 1982). An alternate perspective is to consider the available sample size relative to the number of parameters that the model is estimating. For example, Bentler (1985) recommends at least 5 cases per parameter. We expect to have a sufficiently large sample to permit testing of the causal models described below. Based on the parameters derived from the research described in Section C, we conducted power analyses using Jöreskog and Sörbom's (1993) LISPOWER program. Our analyses evaluated the model's ability to detect the statistical significance of path coefficients that vary in their "true" magnitude, holding the other parameters in the model constant at values derived from the analyses presented in Section C. These power calculations were significantly different from 0 at $p < .05$. We evaluated the statistical power of these models for samples that varied in size from 150 to 170 cases. For the causal paths included in the models discussed in Section C, the power was above .80 for detecting path coefficients of .17 to .20 in absolute magnitude for samples of 150 cases or more. These coefficients correspond to those that Cohen (1988) termed small effects. These results suggest that we will have sufficient power to detect even relatively weak relations among variables.

D6.4 _Testing Hypotheses and Theoretical Models:_

D6.4a _Latent growth modeling (LGM):_ Intervention researchers have begun to examine models that can detect differences between individuals in longitudinal rates of behavioral change. We will, therefore, use growth curve modeling to analyze our data, as they will allow us to examine predictors of change within, as well as across, individuals over time. In addition to standard linear models of growth, a variety of non-linear models (e.g., quadratic, logarithmic, non-parametric) will also be tested. Assumptions of homoscedasticity of residuals will be tested explicitly. After careful examination of the missingness mechanism (Schafer, 1997; Schafer & Graham, 2002), software that can accommodate missing data, such as HLM, AMOS, LISREL, or Mplus, will be used to estimate model parameters. This growth curve model allows for differences between individuals in rates of change over time. Because we are interested primarily in assessing differences at follow-up, the intercept will be centered around post-test or follow-up depending on the specific hypothesis being tested. Based on Muthén and Curran’s (1997) results, our power to detect effects sizes of .2 or greater with an alpha of .05 is expected to exceed .8 with this sample size and a relatively balanced design. Estimates with ANCOVA also suggest power to detect significant differences at this level (Rausch, Maxwell, & Kelley, 2003).

Several important issues must be considered that, due to space limitations, cannot be addressed in detail. First, research on LGM with substance use suggests that an S-shaped logistic function may be more appropriate than a linear function (see McArdle, 2002; Spoth, Redmond, Shin, & Azevedo, 2004). Second, as in any intervention study, the missing data mechanism must be integrated into the analytic plan while considering patterns commonly observed in a mixture model. For example, the groups may include participants engaging in low levels of substance use at the beginning of the study who do not increase their use, early initiators who maintain their initial use levels, participants who increase use gradually, and so forth. We will address this issue by integrating the missing data mechanism (e.g., Hedeker & Gibbons, 1997) and the various types of trajectories (e.g., Muthén, 1997; Nagin & Tremblay, 2001) into a single integrated analytic framework. In addition to this approach, we will also estimate random effects Tobit models using Stata. This allows for the censoring of substance use at 0. Nonusers may vary in the strength of their avoidance of usage; some may be seriously considering trying a particular substance whereas others have no intention whatsoever of using. We will evaluate these models in comparison with others appropriate for our outcome measures, including zero-inflated Poisson and negative binomial models (Long, 2003).

D6.4b. *Aim 5: Testing mediational paths in the causative model:* With our proposed sample size and design, we should be able to detect an effect size of .20 or greater 80% of the time with an alpha of .05 (Muthén & Curran,1997). The sample is larger than needed for detection of significant group differences because we also plan to contribute to theory development by testing mediational pathways; proposing a framework for exploratory analyses; examining moderators of change; comparing various developmental trajectories; and identifying all relevant individual, intervention-related, and time-varying covariates in our analyses.

G. LITERATURE CITED

Agnew, R. (2001). Building on the foundation of general stress theory: Specifying the types of strain most likely to lead to crime and delinquency. *Journal of Research in Crime and Delinquency, 38,* 319-361.

Allison, P. D. (1987). Estimation of linear models with incomplete data. In C. C. Clogg (Ed.), *Sociological methodology* (pp. 71-103). San Francisco: Jossey-Bass.

Anderson, E. (1990). *Streetwise: Race, class, and change in an urban community.* Chicago: University of Chicago Press.

Anthony, J. C., & Petronis, K. R. (1995). Early-onset drug use and risk of later drug problems. *Drug and Alcohol Dependence, 40,* 9-15.

Aquilino, W. S. (1997). From adolescent to young adult: A prospective study of parent-child relations during the transition to adulthood. *Journal of Marriage and the Family, 59*, 670-686.

Arbuckle, J. L. (1996). Full information estimation in the presence of incomplete data. In G. A. Marcoulides & R. E. Schumacker (Eds.), *Advanced structural equation modeling: Issues and techniques* (pp. 243-277). Mahwah, NJ: Erlbaum.

Arnett, J. J. (2000). Emerging adulthood: A theory of development from the late teens through the twenties. *American Psychologist, 55,* 469-480.

Bagley, S. P., Angel, R., Dilworth-Anderson, P., Liu, W., & Schinke, S. (1995). Adaptive health behavior among ethnic minorities. *Health Psychology, 14,* 632-640.

Bandura, A. (1997). *Self-efficacy: The exercise of control.* New York: Freeman.

Bank, L., Dishion, T., Skinner, M., & Patterson, G. R. (1990). Method variance in structural equation modeling: Living with "glop". In G. R. Patterson (Ed.), *Depression and aggression in family interaction* (pp. 247-268). Hillsdale, NJ: Erlbaum.

Barkley, R. A. (1997). Behavioral inhibition, sustained attention, and executive functions: Constructing a unifying theory of ADHD. *Psychological Bulletin, 121,* 65-94.

Bauman, K. E., & Ennett, S. T. (1994). Peer influence on adolescent drug use. *American Psychologist, 49,* 820-822.

Baumeister, R. F., & Sher, S. J. (1988). Self-defeating behavior patterns among normal individuals: Review and analysis of common self-destructive tendencies. *Psychological Bulletin, 104,* 3-22.

Biafora, F., & Zimmerman, R. (1998). Developmental patterns of African-American adolescent drug use. In W. A. Vega & A. G. Gil (Eds.), *Drug use and ethnicity in early adolescence.* New York: Plenum Press.

Boatright, S. R., & Bachtel, D. C. (2003). *The Georgia county guide* (22nd ed.). Athens, GA: University of Georgia Cooperative Extension Service.

Brody, G. H., Flor, D. L., & Gibson, N. M. (1999). Linking maternal efficacy beliefs, developmental goals, parenting practices, and child competence in rural single-parent African American families. *Child Development, 70,* 1197-1208.

Brody, G. H., Flor, D. L., Hollett-Wright, N., & McCoy, J. K. (1998). Children’s development of alcohol use norms: Contributions of parent and sibling norms, children’s temperaments, and parent-child discussions. *Journal of Family Psychology, 12,* 209-219.

Brody, G. H., Flor, D. L., & Neubaum, E. (1998). Coparenting processes and child competence among rural African American families. In M. Lewis & C. Feiring (Eds.), *Families, risk, and competence* (pp. 227-243). Mahwah, NJ: Erlbaum.

Brody, G. H., & Ge, X. (2001). Linking parenting processes and self-regulation to psychological functioning and alcohol use during early adolescence. *Journal of Family Psychology, 15,* 82-94.

Brody, G. H., Ge, X., Conger, R., Gibbons, F. X., Murry, V. M., Gerrard, M., & Simons, R. L. (2001). The influence of neighborhood disadvantage, collective socialization, and parenting on African American children’s affiliation with deviant peers. *Child Development, 72,* 1231-1246.

Brody, G. H., Ge, X., Kim, S. Y., Murry, V. M., Simons, R. L., Gibbons, F. X., Gerrard, M., & Conger, R. (2003). Neighborhood disadvantage moderates associations of parenting and older sibling problem attitudes and behavior with conduct disorders in African American children. *Journal of Consulting and Clinical Psychology, 71,* 211-222.

Brody, G. H., Jack, L., Jr., Murry, V. M., Landers-Potts, M., & Liburd, L. (2001). Heuristic model linking contextual processes to self-management in African American adults with Type 2 diabetes. *Diabetes Educator, 27*(5), 17-25.

Brody, G. H., Kim, S., Murry, V. M., & Brown, A. C. (2004). Protective longitudinal paths linking child competence to behavioral problems among African American siblings. *Child Development, 75*(2).

Brody, G. H., Murry, V. M., Kim, S., & Brown, A. C. (2002). Longitudinal pathways to competence and psychological adjustment among African American children living in rural single-parent households. *Child Development, 73,* 1505-1516.

Brody, G. H., Neubaum, E., Boyd, G. M., & Dufour, M. (1997). Health consequences of alcohol use in rural America. In E. B. Robertson, Z. Sloboda, G. M. Boyd, L. Beatty, & N. J. Kozel (Eds.), *Rural substance abuse: State of knowledge and issues* (NIDA Research Monograph 168, pp. 137-174). Rockville, MD: U.S. Department of Health and Human Services.

Brody, G. H., Stoneman, Z., & Flor, D. (1996a). Family wages, family processes, and youth competence in rural married African-American families. In E. M. Hetherington & E. A. Blechman (Eds.), *Stress, coping, and resiliency in children and families* (pp. 173-188). Hillsdale, NJ: Erlbaum.

Brody, G. H., Stoneman, Z., & Flor, D. (1996b). Parental religiosity, family processes, and youth competence in rural, two-parent African American families. *Developmental Psychology, 32,* 696-706.

Brody, G. H., Stoneman, Z., Flor, D., & McCrary, C. (1994a). Religion's role in organizing family relationships: Family process in rural, two-parent African American families. *Journal of Marriage and the Family, 56,* 878-888.

Brody, G. H., Stoneman, Z., Flor, D., McCrary, C., Hastings, L., & Conyers, O. (1994b). Financial resources, parent psychological functioning, parent co-caregiving, and early adolescent competence in rural two-parent African-American families. *Child Development, 65,* 590-605.

Brown, F., & Tooley, J. (1989). Alcoholism in the Black community. In G. W. Lawson & A. W. Lawson (Eds.), *Alcoholism and substance abuse in special populations.* Rockville, MD: Aspen.

Brown, J. M., Miller, W. R., & Lawendowski, L. A. (1999). The self-regulation questionnaire. *Innovations in clinical practice: A source book* (Vol. 17, pp. 281-292).

Catalano, R. F., Morrison, D. M., Wells, E. A., & Gilmore, M. R. (1992). Ethnic differences in family factors related to early drug initiation. *Journal of Studies on Alcohol, 53,* 208-217.

Chassin, L., Presson, C. C., Pitts, S. C., & Sherman, S. J. (2000). The natural history of cigarette smoking from adolescence to adulthood in a midwestern community sample: Multiple trajectories and their psychosocial correlates. *Health Psychology, 19,* 223-231.

Cole, D. A., & Carpentieri, S. (1990). Social status and comorbidity of child depression and conduct disorder. *Journal of Consulting and Clinical Psychology, 58,* 748-757.

Conger, R. D., & Elder, G. H. (1994). *Families in troubled times: Adapting to changes in rural America.* New York: Aldine de Gruyter.

Conger, R. D., Ge, X., G H Elder Jr., Lorenz, F. O., & Simons, R. L. (1994). Economic stress, coercive family process and developmental problems of adolescents. *Child Development, 65,* 541-561.

Cooper, M. L., Russell, M., & George, W. H. (1988). Coping, expectancies, and alcohol use: Moderating effects of gender, coping, and alcohol expectancies. *Journal of Abnormal Psychology, 101,* 139-152.

Dalaker, J. (2001, September). *Poverty in the United States, 2000* (U.S. Census Bureau Current Population Reports Series P60-214). Washington, DC: U.S. Government Printing Office.

Davey, A. (2001). An analysis of incomplete data. In A. G. Sayer & L. M. Collins (Eds.), *New methods for the analysis of change* (pp. 379-383). Washington, DC: American Psychological Association.

Davey, A., Savla, J. S., & Luo, Z. (2004).*Evaluating model fit with direct maximum likelihood techniques.* Manuscript submitted for publication.

Davey, A., Shanahan, M. J., & Schafer, J. L. (2001). Using multiple imputation to correct for selective attrition in the National Longitudinal Study of Youth. *Journal of Human Resources, 36,* 500-519.

Derogatis, L. R., & Melisaratos, N. (1983). The Brief Symptom Inventory: An introductory report. *Psychological*

Medicine, 13, 595-605.

Dishion, T. J., McCord, J., & Poulin, F. (1999). When interventions harm: Peer groups and problem behavior. *American Psychologist, 54,* 755-764.

Duncan, S. C., Strycker, L. A., & Duncan, T. E. (1999). Exploring associations in developmental trends of adolescent substance use and risky behavior in a high-risk population. *Journal of Behavioral Medicine, 22,* 21-24.

Elder, G. H., Jr. (1992). Life course. In E. Borgatta & M. Borgatta (Eds.), *Encyclopedia of sociology.* New York: Macmillan.

Folkman, S., Chesney, M. A., Pollack, L., & Phillips, C. (1992). Coping and high-risk sexual behavior. *Health Psychology, 11,* 218-222.

Fromme, K., & Rivet, K. (1994). Young adults' coping style as a predictor of their alcohol use and response to daily events. *Journal of Youth and Adolescence, 23,* 85-97.

Frone, M. R., Cooper, M. L., & Russell, M. (1994). Stressful life events, gender, and substance use: An application of Tobit regression. *Psychology of Addictive Behaviors, 8,* 59-69.

Frone, M. R., & Windle, M. (1997). Job dissatisfaction and substance use among employed high school students: The moderating influence of avoidant coping styles. *Substance use and misuse, 32,* 571-585.

Gibbons, F. X., Gerrard, M., Cleveland, M. J., Wills, T. A., & Brody, G. H. (in press). Perceived discrimination and substance use in African American parents and their children: A panel study. *Journal of Personality and Social Psychology.*

Gibbons, F. X., Gerrard, M., & Lane, D. (2003). A social reaction model of adolescent health risk. In J. Suls & K. A. Wallston (Eds.), *Social psychological foundations of health and illness* (pp. 107-136). Malden, MA: Blackwell.

Gibbons, F. X., Gerrard, M., Van de Lune, L. S., Wills, T. A., Brody, G. H., & Conger, R. D. (in press). Context and cognition: Environmental risk and substance use in African American adolescents. *Personality and Social Psychology Bulletin.*

Gillmore, M. R., Catalano, R. F., Jr., Morrison, D. M., & Wells, E. A. (1990). Racial differences in acceptability and availability of drugs and early initiation of substance use. *American Journal of Drug and Alcohol Abuse, 16,* 185-206.

Goldscheider, F. K., & Goldscheider, C. (1997). The historical trajectory of the Black family: Ethnic differences in leaving home over the twentieth century. *History of the Family, 2*, 295-307.

Griesler, P. C., & Kandel, D. B. (1998). Ethnic differences in correlates of adolescent cigarette smoking. *Journal of Adolescent Health, 23,* 167-180.

Guyll, M., Spoth, R., & Redmond, C. (2003). The effects of incentives and research requirements on participation rates for a community-based preventive intervention research study. *Journal of Primary Prevention, 24*, 25-41.

Harper, F. D. (1980). Research and treatment with Black alcoholics. *Alcohol Health and Research World, 4,* 10-16.

Harrison, P. A., Fulkerson, J. A., & Beebe, T. J. (1998). DSM-IV substance use disorder criteria for adolescents: A critical examination based on a statewide school survey. *American Journal of Psychiatry, 155,* 486-492.

Hawkins, J. D., Catalano, R. F., & Miller, J. Y. (1992). Risk and protective factors for alcohol and other drug problems in adolescence and early adulthood: Implications for substance abuse prevention. *Psychological Bulletin, 112,* 64-105.

Heeringa, S.G., & W. L. Rodgers (1998, November). *Information loss in longitudinal surveys: Types, patterns, simple compensation methods.* Workshop presented at the annual meeting of the Gerontological Society of America, Philadelphia, PA.

Herd, D. (1989). *The epidemiology of drinking patterns and alcohol related problems among U.S. Blacks.* Washington, DC: U.S. Government Printing Office.

Holz, V. J., & Tienda, M. (1998). Education and employment in a diverse society: Generating inequality through the school to work transition (pp. 249-281). In N. Denton & S. Tolnay (Eds.), *American diversity: A demographic challenge for the twenty-first century.* Albany, NY: SUNY Press.

Hughes, D., & Chen, L. A. (1999). The nature of parents' race-related communications to children: A developmental perspective. In L. Bolter & C. S. Tamis-LeMonda (Eds.), *Child psychology: A handbook*

of contemporary issues. Philadelphia: Psychology Press.
Institute of Medicine. (1994). *Reducing risks for mental disorders: Frontiers for preventive intervention research.* Washington, DC: National Academy Press.
Jackson, J. S., & Sellars, S. Z. (1997). Psychological, social, and cultural perspectives on minority health in adolescence: A life-course framework. In D. K. Wilson, J. R. Rodriguez, & W. C. Taylor (Eds.), *Health-promoting and health-compromising behavior among minority adolescents.* Washington, DC: American Psychological Association.
Johnston, L. D., O'Malley, P. M., & Bachman, J. G. (2003). *Monitoring the Future national survey results on drug use, 1975-2002. Volume I: Secondary school students* (NIH Publication No. 03-5375). Bethesda, MD: National Institute on Drug Abuse.
Jones-Webb, R. J., Hsiao, C. Y., & Hannan, P. (1995). Relationships between socioeconomic status and drinking problems among Black and White men. *Alcoholism: Clinical and experimental research, 19,* 623-627.
Kandel, D. B. (1995). Ethnic differences in drug use. In G. J. Botvin, S. Schinke, & M. A. Orlandi (Eds.), *Drug abuse prevention with multiethnic youth* (pp. 81-105). Thousand Oaks, CA: Sage.
Kandel, D. B., & Raveis, V. H. (1989). Cessation of illicit drug use in young adulthood. *Archives of General Psychiatry, 46*, 109-116.
Kellam, S. G., & Anthony, J. C. (1998). Targeting early antecedents to prevent tobacco smoking: Findings from an epidemiologically based randomized field trial. *American Journal of Public Health, 88,* 1490-1495.
Kellam, S. G., Ensminger, M. E., & Simon, M. B. (1980). Mental health in first grade and teenage drug, alcohol, and cigarette use. *Drug and Alcohol Dependence, 5,* 273-304.
Klesges, R. C., & Robinson, L. A. (1995). Predictors of smoking onset in adolescent African American boys and girls. *Journal of Health Education, 26,* 85-91.
Krohn, M. D., & Thornberry, T. P. (1993). *Network theory: A model for understanding drug abuse among African American and Hispanic youth* (NIDA Research Monograph 130).
Kurdek, L. A. (1994). Conflict resolution styles in gay, lesbian, heterosexual nonparent, and heterosexual parent couples. *Journal of Marriage & the Family, 56*(3), 705.
Labouvie, E. (1996). Maturing out of substance use: Selection and self-correction. *Journal of Drug Issues, 26,* 457-476.
Leonard, K. E., & Das Eiden, R. (1999). Husband's and wife's drinking: Unilateral or bilateral influences among newlyweds in a general population sample. *Journal of Studies on Alcohol*(Suppl. 13), 130-138.
Leonard, K. E., & Mudar, P. J. (2000). Alcohol use in the year before marriage: Alcohol expectancies and peer drinking as proximal influences on husband and wife alcohol involvement. *Alcoholism Clinical and Experimental Research, 24,* 1666-1679.
Little, R. J. A. (1995).Modeling the drop-out mechanism in repeated-measures studies. *Journal of the American Statistical Association, 90,* 1112-1121.
Little, R. J. A., & Rubin, D. B. (1987).*Statistical analysis with missing data.* New York: Wiley.
Little, R. J. A., & Rubin, D. B. (1989).The analysis of social science data with missing values. *Sociological Methods and Research, 18,* 292-326.
Loeber, R. (1988). Natural histories of conduct problems, delinquency, and associated substance use: Evidence for developmental progressions. In B. B. Lahey & A. E. Kazdin (Eds.), *Advances in clinical child psychology* (Vol. 11, pp. 73-124). New York: Plenum Press.
Loveland-Cherry, C. J., Ross, L. T., & Kaufman, S. R. (1999). Effects of a home-based family intervention on adolescent alcohol use and misuse. *Journal of Studies on Alcohol,*(Suppl. 13), 94-102.
Marini, M. M., Olsen, A. R., & Rubin, D. B. (1980). Maximum likelihood estimation in panel studies with missing data. In K. F. Schuessler (Ed.), *Sociological methodology* (pp. 314-357).San Francisco: Jossey-Bass.
McArdle, J. J. (1994). Structural factor analysis experiments with missing data. *Multivariate Behavioral Research, 29,* 409-454.
McLaughlin, D. K., & Sachs, C. (1988). Poverty in female headed households: Residential differences. *Rural Sociology, 53,* 287-306.
Meng, X. L., & Rubin, D. B. (1992).Performing likelihood ratio tests with multiply-imputed data sets. *Biometrika, 79,* 103-111.
Moffitt, T. E. (1993). Adolescence-limited and life-course persistent antisocial behavior: A developmental

taxonomy. *Psychological Review*, *100*, 674-701.

Moffitt, T. E., Caspi, A., Harrington, H., & Milne, B. J. (2002). Males on the life-course-persistent and adolescence-limited antisocial pathways: Follow-up at age 26 years. *Development and Psychopathology, 14,* 179-207.

Moos, R. H. (1997). Coping Responses Inventory: A measure of approach and avoidance coping skills. In C. P. W. Zalaquett, Richard John (Ed.), *Evaluating stress: A book of resources* (pp. 51-65).

Moos, R. H. (1993). *Coping Responses Inventory Adult Form: Professional manual*. Lutz, FL: Psychological Assessment Resources, Inc.

Murry, V. M. (2000). Extraordinary challenges and ordinary life experiences of Black American families. In P. C. McKenry & S. H. Price (Eds.), *Family stress and change* (2nd ed., pp. 333-358). Thousand Oaks, CA: Sage.

Murry, V. M., & Brody, G. H. (1999). Self-regulation and self-worth of Black children reared in economically stressed, rural, single mother-headed families: The contribution of risk and protective factors. *Journal of Family Issues, 20,* 456-482.

Murry, V. M., & Brody, G. H. (2002). Racial socialization processes in single-mother families: Linking maternal racial identity, parenting, and racial socialization in rural, single-mother families with child self-worth and self-regulation. In H. P. McAdoo (Ed.), *Black children: Social, educational, and parental environments* (2nd ed., pp. 97-115). Thousand Oaks, CA: Sage.

Muthén, B., Kaplan, D., & Hollis, M. (1987). On structural equation modeling with data that are not missing completely at random. *Psychometrika, 52,* 431-462.

Office of Applied Studies. (2003). *Results from the 2002 National Survey on Drug Use and Health: Summary of national findings* (DHHS Publication No. SMA 03-3836, NHSDS Series H-22). Rockville, MD: Substance Abuse and Mental Health Services Administration.

Patterson, G. R., & Yoerger, K. (1997). A developmental model for late-onset delinquency. In D. W. Osgood (Ed.), *Motivation and delinquency: Nebraska Symposium on Motivation* (Vol. 44, pp. 119-177). Lincoln: University of Nebraska Press.

Perry, C. L., Williams, C. L., Veblen-Mortenson, S., Toomey, T. L., Komro, K. A., Anstine, P. S., et al. (1996). Project Northland: Outcomes of a community-wide alcohol use prevention program during early adolescence. *American Journal of Public Health, 86,* 956-965.

Piersma, H. L., Boes, J. L., & Reaume, W. M. (1994). The Brief Symptom Inventory as an outcome measure for adolescent psychiatric inpatients. *Assessment, 1,* 151-158.

Robins, L. N., & Przybeck, T. R. (1985). Age of onset of drug use as a factor in drug and other disorders. In C. L. Jones & R. J. Battjes (Eds.), *Etiology of drug abuse: Implications for Prevention* (pp. 178-192). National Institute on Drug Abuse Research Monograph, Series 56. Washington, DC: U. S. Government Printing Office.

Robins, L. N., & Rutter, M. (Eds.). (1990). *Straight and devious pathways from childhood to adulthood.* New York: Cambridge University Press.

Ross, H. E., Glaser, F. B., & Stiasny, S. (1988). Sex differences in the prevalence of psychiatric disorders in patients with alcohol and drug problems. *British Journal of Addiction, 83,* 1179-1192.

Rubin, D. B. (1987). *Multiple imputation for nonresponse in surveys.* New York: Wiley.

Sampson, R. J., & Lauritsen, J. L. (1994). Violent victimization and offending: Individual, situational, and community-level risk factors. In A. J. Reiss & J. A. Roth (Eds.), *Understanding and preventing violence* (Vol. 3, pp. 1-114). Washington, DC: National Academy Press.

Sanford, M., Offord, D., McLeod, K., Boyle, M., Byrne, C., & Hall, B. (1994). Pathways into the work force: Antecedents of school and work force status. *Journal of the American Academy of Child and Adolescent Psychiatry, 33,* 1036-1046.

Schafer, J. L. (1997) *Analysis of incomplete multivariate data.* London, UK: Chapman & Hall.

Schafer, J.L. & Olsen, M.K. (1998) Multiple imputation for multivariate missing-data problems: A data analyst's perspective. *Multivariate Behavioral Research, 33,* 545–571.

Seligman, M. E. P. (1991). *Learned optimism.* New York: Simon & Shuster.

Semple, S. J., Patterson, T. L., & Grant, I. (2000). The sexual negotiation behavior of HIV-positive gay and bisexual men. *Journal of Consulting and Clinical Psychology, 68,* 934-937.

Sher, K. J., & Trull, T. J. (1994). Personality and disinhibitory psychopathology: Alcoholism and antisocial

personality disorder. *Journal of Abnormal Psychology, 103,* 92-102.

Simons, R. L., Lin, K. H., Gordon, L. C., Brody, G. H., Murry, V. M., & Conger, R. D. (2002). Community differences in the association between parenting practices and child conduct problems. *Journal of Marriage and Family, 64,* 331-345.

Smith, E. P., & Brookins, C. C. (1997). Toward the development of an ethnic identity measure for African American youth. *Journal of Black Psychology, 23,* 358-377.

Spielberger, C. D., Johnson, E. H., Russel, S. F., Crane, R. J., Jacobs, G. A., & Worden, T. J. (1985). The experience and expression of anger: Construction and validation of an anger expression scale. In M. A. Chesney & R. H. Rosenman (Eds.), *Anger and hostility in cardiovascular and behavioural disorders* (pp. 5-30). New York: Hemisphere.

Spoth, R., Redmond, C., & Lepper, H. (1999a). Alcohol initiation outcomes of universal family-focused preventive interventions: One- and two-year follow-ups of a controlled study. *Journal of Studies on Alcohol*(Suppl. 13), 103-111.

Spoth, R., Reyes, M. L., Redmond, C., & Shin, C. (1999). Assessing a public health approach to delay onset and progression of adolescent substance use: Latent transition and log-linear analyses of longitudinal family preventive intervention outcomes. *Journal of Consulting and Clinical Psychology, 67,* 619-630.

Stevenson, H. C. (1997). Validation of the Scale of Racial Socialization for African American Adolescents: Steps toward multidimensionality. *Journal of Black Psychology, 20,* 445-468.

Stevenson, H. C., Reed, J., Bodison, P., & Bishop, A. (1997). Racism stress management: Racial socialization beliefs and the experience of depression and anger in African American youth. *Youth and Society, 29,* 197-222.

Struss, D. T. (1992). Biological and psychological development of executive functions. *Brain and Cognition, 20,* 8-23.

Tickamyer, A. R., & Duncan, C. M. (1990). Poverty and opportunity structure in rural America. *Annual Review of Sociology, 16,* 67-86.

Vega, W. A., Zimmerman, R. S., Warheit, G. J., Apospori, E., & Gil, A. G. (1993). Risk factors for early adolescent drug use in four ethnic and racial groups. *American Journal of Public Health, 83,* 185-189.

Walker, S., Spohn, C., & DeLone, M. (2000). *The color of justice: Race, ethnicity, and crime in America* (2nd ed.). Belmont, CA: Wadsworth.

Wallace, J. M., Jr. (1994). Race differences in adolescent drug use: Recent findings from national samples. *African American Research Perspectives, 1,* 31-55.

Wallace, J. M., Jr. (1999). The social ecology of addiction: Race, risk, and resilience. *Pediatrics, 103,* 1122-1127.

Wallace, J. M., Jr., & Bachman, J. G. (1991). Explaining racial/ethnic differences in adolescent drug use: The impact of background and lifestyle. *Social Problems, 38,* 333-357.

Wallace, J. M., Jr., Bachman, J. G., O'Malley, P. A., & Johnston, L. D. (1995). Racial/ethnic differences in adolescent drug use. In G. Botvin, S. Schinke, & M. Orlandi (Eds.), *Drug abuse prevention with multiethnic youth* (pp. 59-80). Thousand Oaks, CA: Sage.

Wallace, J. M., Jr., Bachman, J. G., O'Malley, P. M., Johnston, L. D., Schulenberg, J. E., & Cooper, S. M. (2002). Tobacco, alcohol, and illicit drug use: racial and ethnic differences among U.S. high school seniors, 1976-2000. *Public Health Reports, 117*(Suppl. 1), S67-75.

Welte, J., & Barnes, G. (1987). Alcohol use among adolescent minority groups. *Journal of Studies on Alcohol, 48,* 329-336.

White, H. R., & Labouvie, E. W. (1989). Towards the assessment of adolescent problem drinking. *Journal of Studies on Alcohol, 50,* 30-37.

White, H. R., Pandina, R. J., & Chen, P.-H. (2002). Developmental trajectories of cigarette use from early adolescence into young adulthood. *Drug and Alcohol Dependence, 65,* 167-178.

Willis, W. (1992). Families with African American roots. In E. W. Lynch & M. J. Hansons (Eds.), *Developing cross-cultural competence: A guide for working with young children and their families* (pp. 121-150). Baltimore: Brookes.

Wills, T. A., Gibbons, F. X., Gerrard, M., & Brody, G. H. (2000). Protection and vulnerability processes relevant for early onset of substance use: A test among African-American children. *Health Psychology, 19,* 253-263.

Wills, T. A., Gibbons, F. X., Gerrard, M., Murry, V. M., & Brody, G. H. (2003). Family communication and religiosity related to substance use and sexual behavior in early adolescence: A test for pathways through self-control and prototype perceptions. *Psychology of Addictive Behaviors, 17,* 312-323.
Wills, T. A., Sandy, J. M., & Yaeger, A. M. (2002). Moderators of the relation between substance use level and problems: Test of a self-regulation model in middle adolescence. *Journal of Abnormal Psychology, 111,* 3-21.
Wills, T. A., & Shiffman, S. (1985). Coping and substance abuse: A conceptual framework. In S. Shiffman & T. A. Wills (Eds.), *Coping and substance abuse* (pp. 1-21). New York: Academic Press.
Wills, T. A., & Stoolmiller, M. (2003). The role of self-control in early escalation of substance use: A time-varying analysis. *Journal of Consulting and Clinical Psychology, 114,* 201-212.
Wills, T. A., Windle, M., & Cleary, S. D. (1998). Temperament and novelty seeking in adolescent substance use: Convergence of dimensions of temperament with constructs from Cloninger's theory. *Journal of Personality and Social Psychology, 74,* 387-406.
Young, B. (2003). *Public high school dropouts and completers from the Common Core of Data: School year 2000-1* (Web Document NCES 2004310). Washington D.C.: National Center for Education Statistics, Institute of Educational Science, Department of Education.
Zimbardo, P. G., Keough, K. A., & Boyd, J. N. (1997). Present time perspective as a predictor of risky driving. *Personality and Individual Differences, 23,* 1007-1023.
Zucker, R. A. (1994). Pathways to alcohol problems: A developmental account of the evidence for multiple alcoholisms and contextual contributions to risk. In R. A. Zucker, J. Howard, & G. M. Boyd (Eds.), *The development of alcohol problems* (pp. 255-289). Bethesda, MD: National Institute on Alcohol Abuse and Alcoholism.
