# Supplementary material for: Family-Centered Prevention Effects on the Association Between Racial Discrimination and Mental Health in Black Adolescents: Secondary Analysis of 2 Randomized Clinical Trials
Source: JAMA Netw Open. 2021 Mar 24;4(3):e211964. doi: 10.1001/jamanetworkopen.2021.1964 (PMC7991970; doi:10.1001/jamanetworkopen.2021.1964)
Supplement: Supplement 2. — eTable 1. Correlations and Descriptive Statistics Among Study Variables: SAAF–T eTable 2. Correlations and Descriptive Statistics Among Study Variables: AIM eTable 3. Changes in Protective Caregiving From Baseline to Postintervention and the Effects of Encountered Discrimination on Conduct Problems at Long-Term Follow-up by Intervention Status: SAAF–T eTable 4. Changes in Protective Caregiving From Baseline to Postintervention and the Effects of Encountered Discrimination on Depressive Symptoms at Long-Term Follow-up by Intervention Status: SAAF–T eTable 5. Changes in Protective Caregiving From Baseline to Postintervention and the Effects of Encountered Discrimination on Conduct Problems at Long-Term Follow-up by Intervention Status: AIM eTable 6. Changes in Protective Caregiving From Baseline to Postintervention and the Effects of Encountered Discrimination on Depressive/Anxious Symptoms at Long-Term Follow-up by Intervention Status: AIM eTable 7. Conditional Indirect Effects (Moderated Mediation Effects) for Low and High Encountered Discrimination Conditions eFigure. Estimated Means of Changes in Protective Caregiving From Baseline to Postintervention Conduct Problems and Depressive or Anxious Symptoms [file jamanetwopen-e211964-s002.pdf]

Supplemental Online Content

Brody GH, Yu T, Chen E, Miller GE, Barton AW, Kogan SM. Family-centered prevention effects on the association between racial discrimination and mental health in Black adolescents: secondary analysis of 2 randomized clinical trials. *JAMA Netw Open*. 2021;4(3):e211964. doi:10.1001/jamanetworkopen.2021.1964

eTable 1. Correlations and Descriptive Statistics Among Study Variables: SAAF–T
eTable 2. Correlations and Descriptive Statistics Among Study Variables: AIM
eTable 3. Changes in Protective Caregiving From Baseline to Postintervention and the Effects of Encountered Discrimination on Conduct Problems at Long-Term Follow-up by Intervention Status: SAAF–T
eTable 4. Changes in Protective Caregiving From Baseline to Postintervention and the Effects of Encountered Discrimination on Depressive Symptoms at Long-Term Follow-up by Intervention Status: SAAF–T
eTable 5. Changes in Protective Caregiving From Baseline to Postintervention and the Effects of Encountered Discrimination on Conduct Problems at Long-Term Follow-up by Intervention Status: AIM
eTable 6. Changes in Protective Caregiving From Baseline to Postintervention and the Effects of Encountered Discrimination on Depressive/Anxious Symptoms at Long-Term Follow-up by Intervention Status: AIM
eTable 7. Conditional Indirect Effects (Moderated Mediation Effects) for Low and High Encountered Discrimination Conditions
eFigure. Estimated Means of Changes in Protective Caregiving From Baseline to Postintervention Conduct Problems and Depressive or Anxious Symptoms

This supplemental material has been provided by the authors to give readers additional information about their work.

eTable 1. Correlations and Descriptive Statistics Among Study Variables: SAAF–T

Variable	Mean (*SD*) or n (%)	Correlations								
		1	2	3	4	5	6	7	8	9
1. Sex, male	221 (44.0%)	—								
2. Intervention, SAAF–T	252 (50.2%)	.009	—							
3. Family socioeconomic risk (baseline)	2.321 (1.428)	-.039	.068	—						
4. Encountered discrimination (baseline)	13.054 (4.725)	.077	-.005	-.032	—					
5. Protective caregiving (baseline)	0 (1.703)	-.022	.047	-.160***	.045	—				
6. Protective caregiving (post-intervention)	0 (1.663)	-.041	.065	-.171***	.063	.620***	—			
7. Conduct problems (baseline)	5.173 (8.558)	.094*	-.041	.048	.157***	-.030	-.015	—		
8. Conduct problems (follow-up)	3.536 (6.859)	.099*	-.078	.063	.177***	-.081	-.173***	.463***	—	
9. Depressive symptoms (baseline)	13.803 (8.695)	-.158***	-.001	.070	.247***	-.073	-.004	.245***	.153***	—
10. Depressive symptoms (follow-up)	13.090 (8.865)	-.048	-.077	.091*	.135**	-.093*	-.056	.167***	.226***	.457***

N = 465-502. **p* < .05. ***p* < .01. ****p* < .001.

© 2021 Brody GH et al. *JAMA Network Open.*

eTable 2. Correlations and Descriptive Statistics Among Study Variables: AIM

Variable	Mean (*SD*) or n (%)	Correlations 1	2	3	4	5	6	7	8	9
1. Sex, male	150 (40.9%)	—								
2. Intervention, AIM	187 (51.0%)	-.082	—							
3. Family socioeconomic risk (baseline)	2.014 (1.398)	.024	.010	—						
4. Encountered discrimination (baseline)	4.640 (3.853)	.026	-.062	-.089	—					
5. Protective caregiving (baseline)	53.338 (5.706)	.008	-.026	-.035	-.057	—				
6. Protective caregiving (post-intervention)	54.136 (5.078)	.014	.061	-.041	-.006	.632***	—			
7. Conduct problems (baseline)	4.371 (4.292)	-.003	.059	.089	.140**	-.230***	-.159**	—		
8. Conduct problems (follow-up)	2.790 (3.988)	.001	-.033	.131*	.117*	-.154**	-.238***	.566***	—	
9. Depressive/Anxious symptoms (baseline)	2.515 (3.069)	-.007	.035	.096	.148**	-.176**	-.149**	.604***	.460***	—
10. Depressive/Anxious symptoms (follow-up)	1.506 (2.746)	-.014	-.029	.101	.162**	-.135*	-.209***	.424***	.685***	.602***

N = 300-367. *$p < .05$. **$p < .01$. ***$p < .001$.

© 2021 Brody GH et al. *JAMA Network Open.*

eTable 3. Changes in Protective Caregiving From Baseline to Postintervention and the Effects of Encountered Discrimination on Conduct Problems at Long-Term Follow-up by Intervention Status: SAAF–T

	Model 1			Model 2	
	Post-Intervention Protective Caregiving			Long-Term Follow-Up Conduct Problems	
Predictors	*b*	[95% CI]		*b*	[95% CI]
1. Sex, male	-.104	[-.335, .127]		.243	[-.057, .544]
2. Family socioeconomic risk (baseline)	-.086*	[-.161, -.011]		.055	[-.060, .170]
3. Conduct problems (baseline)	.002	[-.010, .015]		.057***	[.046, .068]
4. Protective caregiving (baseline)	.593***	[.515, .671]		.045	[-.118, .208]
5. Encountered discrimination (baseline)	-.071	[-.213, .071]		.289**	[.112, .467]
6. Intervention, SAAF–T	.145	[-.084, .373]		-.092	[-.412, .228]
7. Encountered discrimination × SAAF–T	.284**	[.094, .475]		-.405**	[-.691, -.119]
8. Protective caregiving (post-intervention)	-	-		-.148*	[-.266, -.030]

N = 502; *b* = unstandardized regression coefficient; CI = confidence interval.

**p* < .05. ***p* < .01. ****p* < .001.

© 2021 Brody GH et al. *JAMA Network Open.*

eTable 4. Changes in Protective Caregiving From Baseline to Postintervention and the Effects of Encountered Discrimination on Depressive Symptoms at Long-Term Follow-up by Intervention Status: SAAF–T

	Model 1			Model 2	
	Post-Intervention Protective Caregiving			Long-Term Follow-Up Depressive Symptoms	
Predictors	*b*	[95% CI]		*b*	[95% CI]
1. Sex, male	-.086	[-.321, .148]		.025	[-.080, .130]
2. Family socioeconomic risk (baseline)	-.094*	[-.175, -.013]		.029	[-.008, .065]
3. Depressive symptoms (baseline)	.075	[-.130, .281]		.493***	[.401, .585]
4. Protective caregiving (baseline)	.601***	[.532, .670]		-.016	[-.055, .023]
5. Encountered discrimination (baseline)	-.070	[-.229, .089]		.032	[-.038, .103]
6. Intervention, SAAF–T	.144	[-.084, .372]		-.103*	[-.205, -.001]
7. Encountered discrimination × SAAF–T	.282*	[.055, .509]		-.032	[-.135, .071]
8. Protective caregiving (post-intervention)	-	-		-.005	[-.045, .035]

N = 502; *b* = unstandardized regression coefficient; CI = confidence interval.

**p* < .05. ***p* < .01. ****p* < .001.

© 2021 Brody GH et al. *JAMA Network Open.*

eTable 5. Changes in Protective Caregiving From Baseline to Postintervention and the Effects of Encountered Discrimination on Conduct Problems at Long-Term Follow-up by Intervention Status: AIM

	Model 1			Model 2	
	Post-Intervention Protective Caregiving			Long-Term Follow-Up Conduct Problems	
Predictors	*b*	[95% CI]		*b*	[95% CI]
1. Sex, male	.055	[-.810, .921]		-.010	[-.161, .142]
2. Family socioeconomic risk (baseline)	-.054	[-.363, .254]		.035	[-.019, .089]
3. Conduct problems (baseline)	-.177	[-.716, .363]		.572***	[.477, .667]
4. Protective caregiving (baseline)	.568***	[.491, .645]		.010	[-.007, .027]
5. Encountered discrimination (baseline)	-.176	[-.766, .413]		.160**	[.052, .267]
6. Intervention, AIM	.774	[-.076, 1.624]		-.087	[-.237, .063]
7. Encountered discrimination × AIM	.890*	[.045, 1.734]		-.228**	[-.379, -.077]
8. Protective caregiving (post-intervention)	-	-		-.029**	[-.049, -.009]

$N = 367$; *b* = unstandardized regression coefficient; CI = confidence interval.

*$p < .05$. **$p < .01$. ***$p < .001$.

eTable 6. Changes in Protective Caregiving From Baseline to Postintervention and the Effects of Encountered Discrimination on Depressive/Anxious Symptoms at Long-Term Follow-up by Intervention Status: AIM

	Model 1			Model 2	
	Post-Intervention Protective Caregiving			Long-Term Follow-Up Depressive/Anxious Symptoms	
Predictors	*b*	[95% CI]		*b*	[95% CI]
1. Sex, male	.049	[-.817, .915]		-.041	[-.169, .088]
2. Family socioeconomic risk (baseline)	-.061	[-.370, .248]		.025	[-.021, .070]
3. Depressive/anxious symptoms (baseline)	-.169	[-.735, .398]		.559***	[.474, .644]
4. Protective caregiving (baseline)	.571***	[.494, .648]		.006	[-.008, .020]
5. Encountered discrimination (baseline)	-.167	[-.759, .426]		.114*	[.022, .205]
6. Intervention, AIM	.773	[-.078, 1.623]		-.075	[-.202, .052]
7. Encountered discrimination × AIM	.856*	[.011, 1.701]		-.113	[-.241, .015]
8. Protective caregiving (post-intervention)	-	-		-.022**	[-.039, -.006]

N = 367; *b* = unstandardized regression coefficient; CI = confidence interval.

**p* < .05. ***p* < .01. ****p* < .001.

© 2021 Brody GH et al. *JAMA Network Open.*

eTable 7. Conditional Indirect Effects (Moderated Mediation Effects) for Low and High Encountered Discrimination Conditions

Conditions		Regression Coefficients			Indirect Effect	
		Intervention status to changes in protective caregiving (path A)	Changes in protective caregiving to mental health outcome (path B)		Estimates (path A × path B)	95% CI
SAAF-T conduct problems						
High encountered discrimination		0.429	-0.148		-0.063	[-0.127, -0.001]
Low encountered discrimination		-0.139	-0.148		0.021	[-0.032, 0.073]
AIM conduct problems						
High encountered discrimination		1.664	-0.029		-0.048	[-0.095, -0.001]
Low encountered discrimination		-0.116	-0.029		0.003	[-0.031, 0.038]
AIM depressive/anxious symptoms						
High encountered discrimination		1.629	-0.022		-0.036	[-0.074, 0]
Low encountered discrimination		-0.083	-0.022		0.002	[-0.025, 0.029]

© 2021 Brody GH et al. *JAMA Network Open.*

eFigure. Estimated Means of Changes in Protective Caregiving From Baseline to Postintervention Conduct Problems and Depressive or Anxious Symptoms

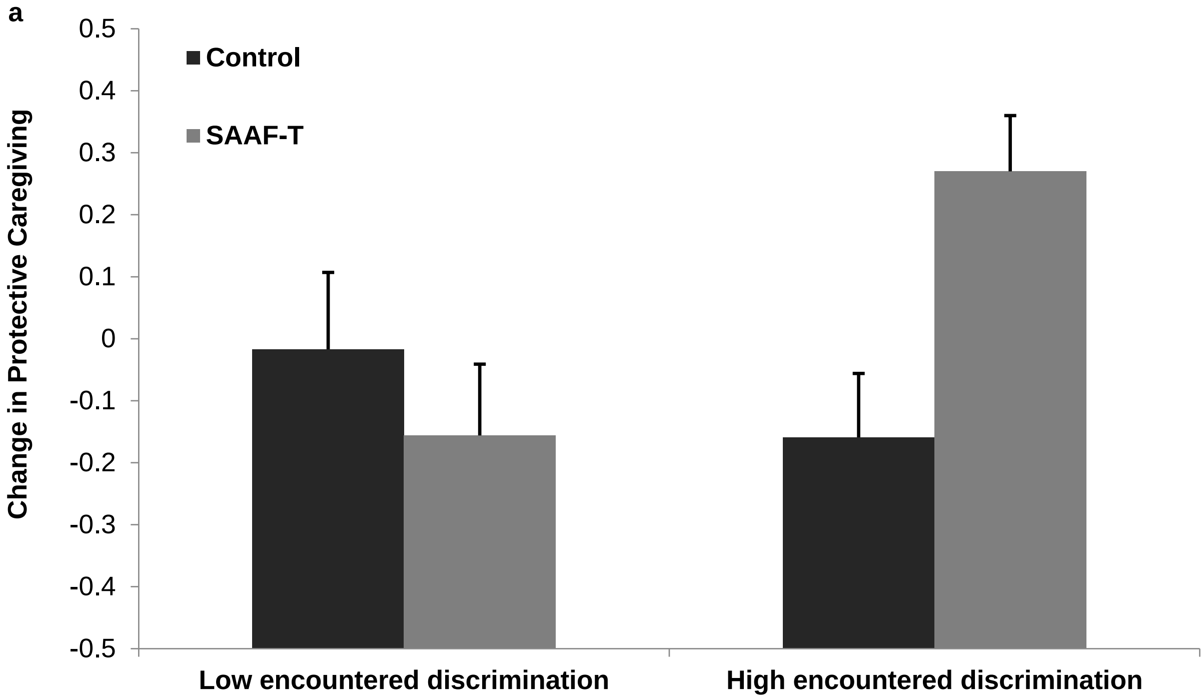

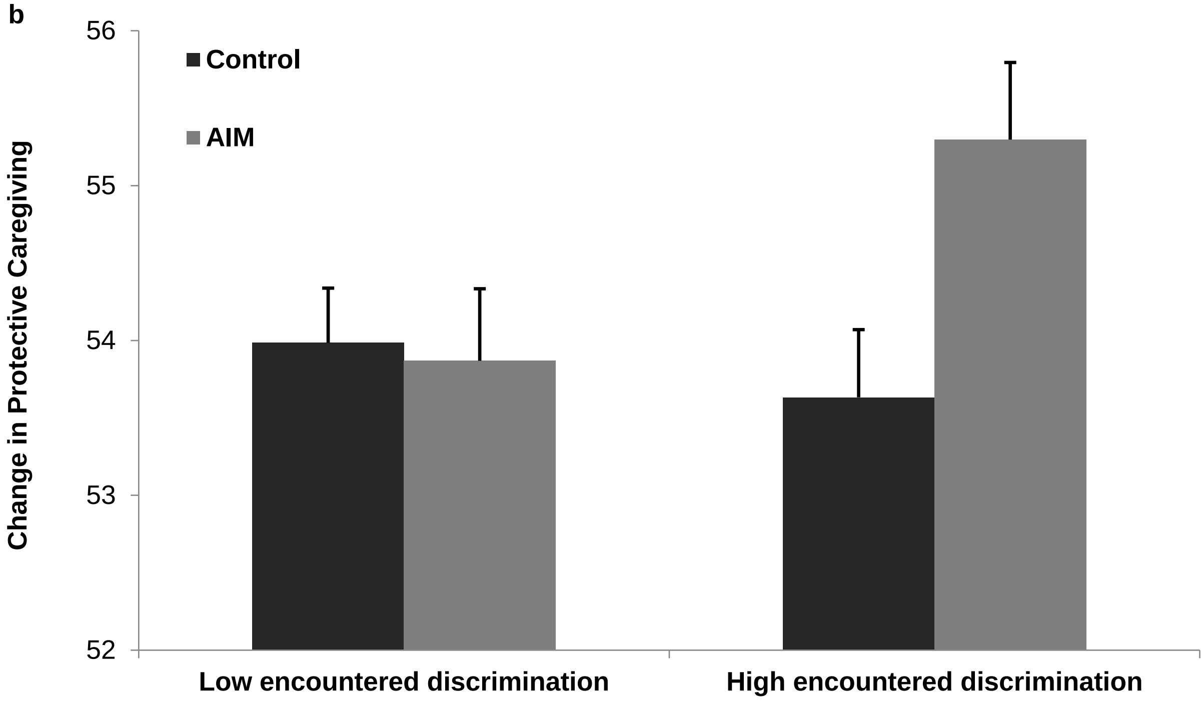

© 2021 Brody GH et al. *JAMA Network Open.*

c

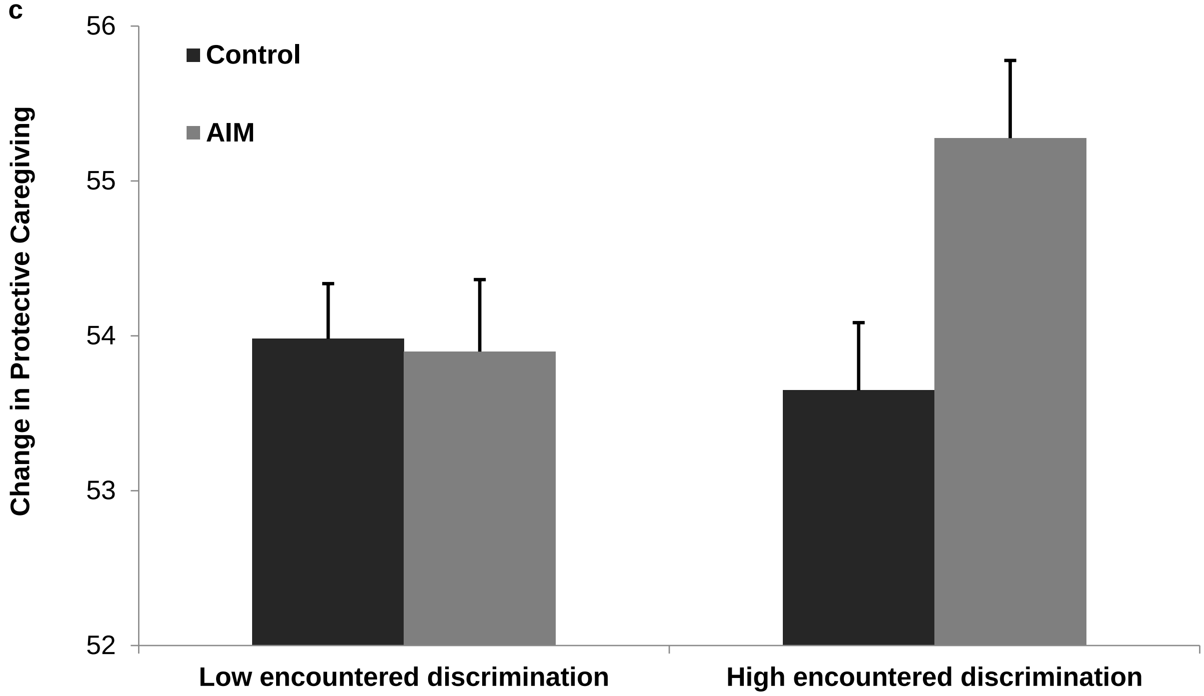

eFigure. Estimated means of changes in protective caregiving from baseline to post-intervention for SAAF-T (a), for AIM with conduct problems as the outcome (b), and for AIM with depressive/anxious symptoms as the outcome (c) for the control and intervention groups at low (-1 *SD*) vs. high (+1 *SD*) levels of encountered racial discrimination at baseline. Error bars = ±1 standard error.

© 2021 Brody GH et al. *JAMA Network Open.*
