# Supplementary material for: Family-Centered Prevention Effects on the Association Between Racial Discrimination and Mental Health in Black Adolescents: Secondary Analysis of 2 Randomized Clinical Trials
Source: JAMA Netw Open. 2021 Mar 24;4(3):e211964. doi: 10.1001/jamanetworkopen.2021.1964 (PMC7991970; doi:10.1001/jamanetworkopen.2021.1964)
Supplement: Supplement 3. — Data Sharing Statement [file jamanetwopen-e211964-s003.pdf]

Brody GH, Yu T, Chen E, et al. Family-centered prevention effects on the association between racial discrimination and mental health in Black adolescents. *JAMA Netw Open*. 2021;4(3):e211964. doi:10.1001/jamanetworkopen.2021.1964

Data Sharing Statement

Data

Data available: No

Additional Information

Explanation for why data not available: The study populations did not consent to any data sharing and they reported on sensitive information.
